# Rapid DNA unwinding accelerates genome editing by engineered CRISPR-Cas9

**DOI:** 10.1016/j.cell.2024.04.031

**Published:** 2024-05-22

**Authors:** Amy R. Eggers, Kai Chen, Katarzyna M. Soczek, Owen T. Tuck, Erin E. Doherty, Bryant Xu, Marena I. Trinidad, Brittney W. Thornton, Peter H. Yoon, Jennifer A. Doudna

**Affiliations:** 1 Department of Molecular and Cell Biology, University of California, Berkeley, Berkeley, CA 94720, USA; 2 Innovative Genomics Institute, University of California, Berkeley, Berkeley, CA 94720, USA; 3 California Institute for Quantitative Biosciences, University of California, Berkeley, Berkeley, CA 94720, USA; 4 Department of Chemistry, University of California, Berkeley, Berkeley, CA 94720, USA; 5 Howard Hughes Medical Institute, University of California, Berkeley, Berkeley, CA 94720, USA; 6 Gladstone Institutes, San Francisco, CA 94158, USA; 7 Gladstone-UCSF Institute of Genomic Immunology, San Francisco, CA 94158, USA; 8 Molecular Biophysics and Integrated Bioimaging Division, Lawrence Berkeley National Laboratory, Berkeley, CA 94720, USA; 9 These authors contributed equally; 10 Lead contact

## Abstract

Thermostable clustered regularly interspaced short palindromic repeats (CRISPR) and CRISPR-associated (Cas9) enzymes could improve genome-editing efficiency and delivery due to extended protein lifetimes. However, initial experimentation demonstrated *Geobacillus stearothermophilus* Cas9 (GeoCas9) to be virtually inactive when used in cultured human cells. Laboratory-evolved variants of GeoCas9 overcome this natural limitation by acquiring mutations in the wedge (WED) domain that produce >100-fold-higher genome-editing levels. Cryoelectron microscopy (cryo-EM) structures of the wild-type and improved GeoCas9 (iGeoCas9) enzymes reveal extended contacts between the WED domain of iGeoCas9 and DNA substrates. Biochemical analysis shows that iGeoCas9 accelerates DNA unwinding to capture substrates under the magnesium-restricted conditions typical of mammalian but not bacterial cells. These findings enabled rational engineering of other Cas9 orthologs to enhance genome-editing levels, pointing to a general strategy for editing enzyme improvement. Together, these results uncover a new role for the Cas9 WED domain in DNA unwinding and demonstrate how accelerated target unwinding dramatically improves Cas9-induced genome-editing activity.

## In brief

A structure-function exploration of an evolved genome editor, iGeoCas9, reveals that the WED domain governs target DNA unwinding speed and drives efficient genome editing.

## Graphical abstract

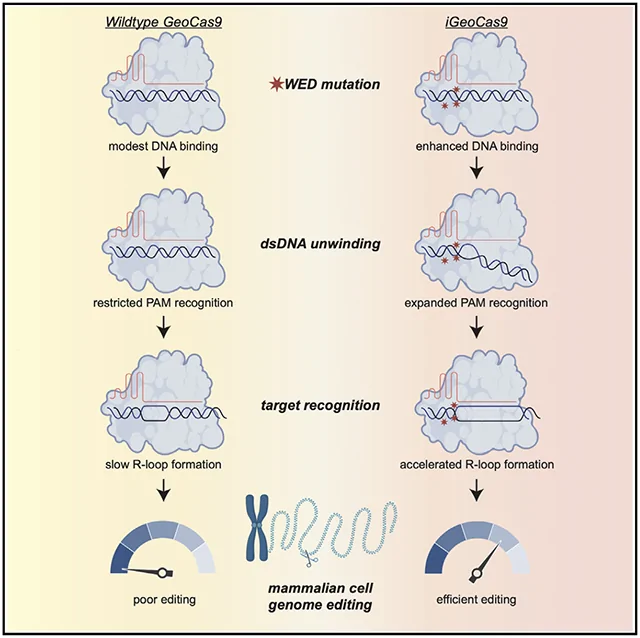

## INTRODUCTION

Clustered regularly interspaced short palindromic repeats (CRISPR) and CRISPR-associated (Cas) proteins provide adaptive immunity for prokaryotes by selectively targeting and cleaving nucleic acids of invading viruses and mobile genetic elements.^1^ This defense mechanism relies on CRISPR RNA-guided Cas proteins to distinguish foreign DNA from endogenous DNA. The ~20-nucleotide (nt) guide sequence in the RNA uses base-pairing complementarity to recognize a foreign DNA target, triggering its Cas-mediated cleavage.^2^ The ease of guide RNA reprogramming is central to applications of CRISPR-Cas systems for genome editing.^3,4^ CRISPR-Cas9 from *Streptococcus pyogenes* (SpyCas9), the most widely adopted editor, is commonly used in research as well as clinical and agricultural applications.^5^ However, its sensitivity to aggregation or proteolytic inactivation can inhibit genome-editing outcomes under some conditions *in vitro* and *in vivo*.^6^

The thermostable enzyme Cas9 from *Geobacillus stearothermophilus* (GeoCas9)^7,8^ was hypothesized to have enhanced genome-editing activity based on extended protein lifetime, but this was not borne out by experimental evidence. Instead, data showed GeoCas9 to be a poor editor in human cells despite its thermostability and robust biochemical activity.^7–9^ The high stability of GeoCas9 made it a candidate to withstand ribonucleoprotein (RNP) delivery to tissues, but poor activity prevented it from being utilized as a robust genome editor. Directed evolution was applied to engineer GeoCas9 based on a bacterial dual-plasmid selection system, yielding variants with improved editing activity and expanded PAM flexibility in cultured cells and in mouse tissues.^9^ The resulting iGeoCas9 enzyme, which shows >100-fold increases in genome-editing efficiencies, contains mutations in the wedge (WED) domain that increase activity while maintaining thermal tolerance and protein stability.^9^

To determine the molecular basis for the dramatic improvement of iGeoCas9 as a genome editor, we used a combination of cryoelectron microscopy (cryo-EM) and biochemical analysis to compare the structures and behaviors of wild-type versus iGeoCas9 enzymes. The three WED-domain mutations responsible for improved editing by iGeoCas9 were found to establish new interactions with target double-stranded DNA (dsDNA), leading to enhanced DNA binding and a relaxed preference for protospacer-adjacent motif (PAM) base pairing next to the target sequence. Using a fluorescent reporter assay, we found that the improved dsDNA binding dramatically accelerated DNA unwinding, expanding the target sequence space by reducing PAM specificity. Furthermore, iGeoCas9 WED-domain mutations enable the enzyme to function at dramatically reduced magnesium ion concentrations consistent with those found in mammalian but not bacterial cells. Similar WED-domain mutations introduced into other Cas9 enzymes also enhanced genome-editing activity in cells, implicating this structural region as a pivotal and previously unappreciated moderator of Cas9 target recognition. Together, these data reveal an unexpected connection between protein-DNA binding and helix unwinding that explains how Cas9 enzymatic activity can be modified to enhance genome editing.

## RESULTS

### GeoCas9 molecular structures reveal unexpected WED domain-DNA contacts

**Figure 1.**
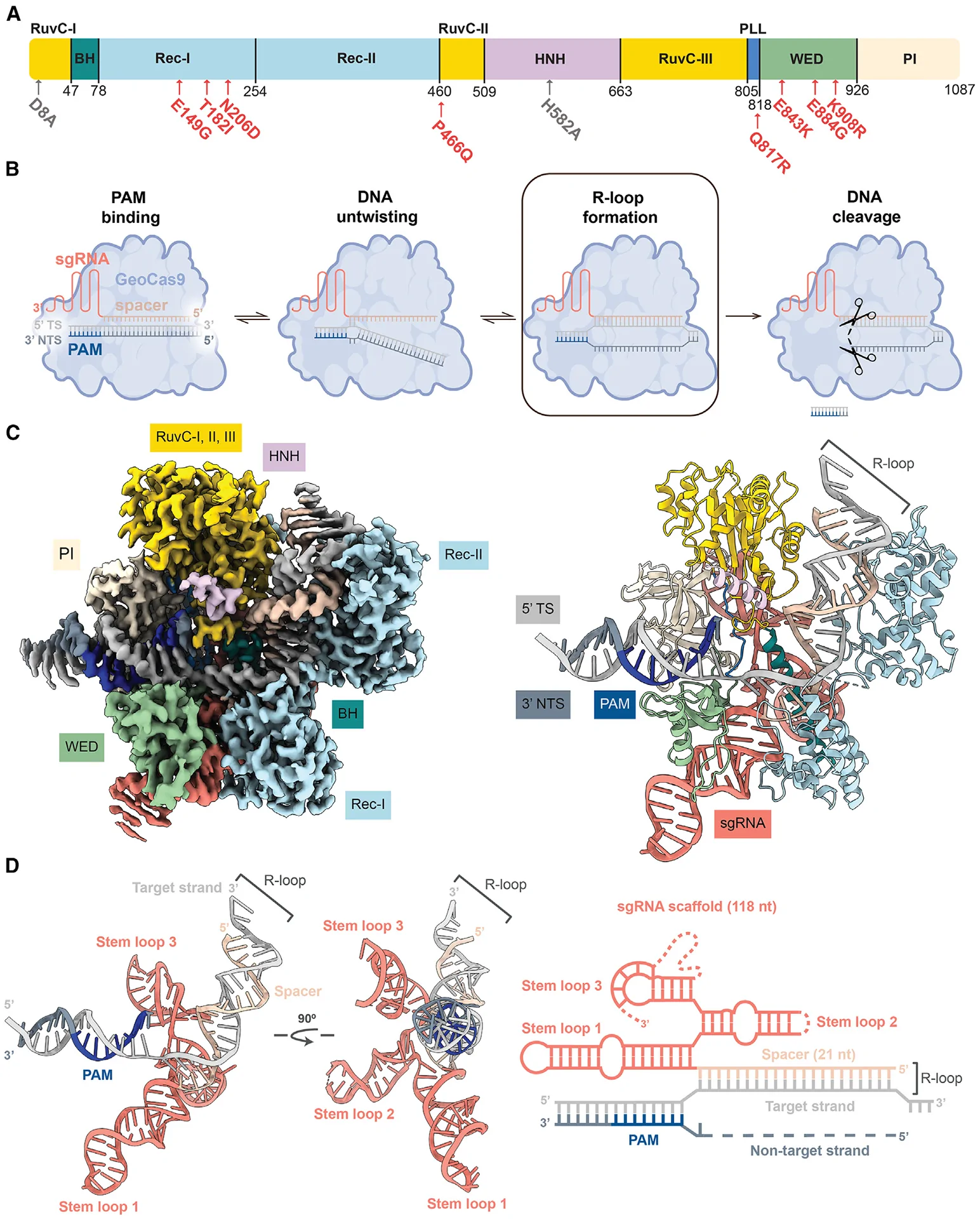
Cryo-EM iGeoCas9-sgRNA-DNA ternary complex (A) Domain organization of GeoCas9. iGeoCas9 mutations indicated with red arrows. Deactivating mutations indicated with gray arrows. BH, bridge helix; WED, wedge; PI, PAM interacting; PLL, phosphate lock loop. (B) CRISPR-Cas9 dsDNA targeting pathway for active enzymes. Deactivated Cas9 concludes at the R-loop formation step. (C) Surface (left) and ribbon (right) representation of iGeoCas9-sgRNA-DNA ternary complex. Domains are colored as in (A). Nucleic acid is colored as in (D). BH, bridge helix; WED, wedge; PI, PAM interacting; TS, target strand; NTS, non-target strand; PAM, protospacer adjacent motif; sgRNA, single guide RNA. (D) Cartoon representation of iGeoCas9 sgRNA-dsDNA complex (left). Schematic representation of iGeoCas9 sgRNA:target DNA complex (right). PAM, protospacer adjacent motif. See also Figures S1–S3 and Table S1.

To understand why iGeoCas9 is an efficient genome editor when its parent enzyme is not, we first explored the structural differences between wild-type GeoCas9 and iGeoCas9. iGeoCas9 contains eight engineered mutations to wild-type GeoCas9 (Figure 1A), including three in the Rec domain (E149G, T182I, and N206D), one in the RuvC domain (P466Q), one in the phosphate lock loop (Q817R), and three in the WED domain (E843K, E884G, and K908R). In order to see how the mutations affect Cas9’s function on target DNA, we reconstituted the ternary structures of catalytically deactivated wild-type GeoCas9 and iGeoCas9 (with nuclease-deactivating mutations, D8A and H582A, introduced to the RuvC and HNH domains, respectively) complexed with single-guide RNA (sgRNA) and target DNA to capture nucleic acid interactions that occur upon R-loop formation (Figure 1B). Cryo-EM reconstructions of wild-type GeoCas9 and iGeoCas9 (3.17 and 2.63Å resolution, respectively) reveal similar domain architecture to other type II-C enzymes, with a smaller REC domain relative to type II-A enzymes (Figures 1C and S1–S3). A full 21-nt target strand (TS) DNA binds the complementary guide RNA sequence and the entire 5′-N_4_CAAA-3′ PAM-containing dsDNA duplex. Only 1 nt of the non-TS (NTS) was resolved, with the remainder disordered. The sgRNA scaffold forms a triple stem-loop architecture, with a nucleotide triplex in stem loop 3; unsharpened maps indicate a possible fourth stem loop, but the density was insufficient to build a reliable model (Figure 1D). Wild-type GeoCas9 and iGeoCas9 structures are overall very similar, both having disordered HNH domains, likely representing the precatalytic states (Figure S3).

**Figure 2.**
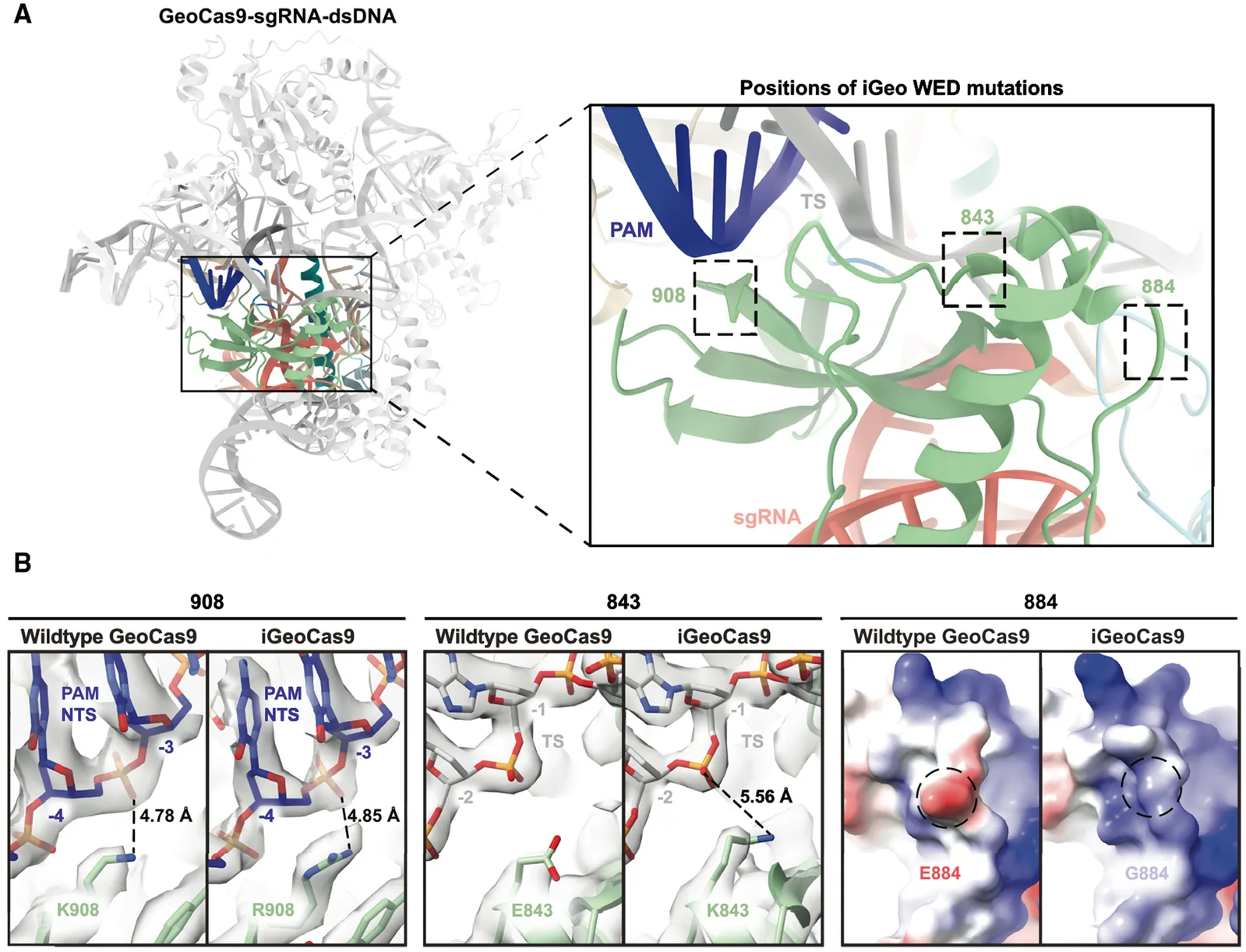
Structural comparison of wild-type GeoCas9 and iGeoCas9 WED domain (A) Ribbon representation of iGeoCas9 WED domain boxed with solid line. Amino acid mutation positions are boxed with a dashed line. Domains are colored as in Figure 1A. Nucleic acid is colored as in Figure 1D. TS, target strand; PAM, protospacer adjacent motif; sgRNA, single guide RNA. (B) Comparison of wild-type GeoCas9 and iGeoCas9 at WED domain amino acid positions 908, 843, and 884. Positions 908 and 843 are represented by an EM density map (gray) and model (sticks and ribbons). PAM nucleotide positions on the non-target strand are labeled in descending from −1 to −8 in the 5′ to 3′ direction. Nucleotides on the target strand are assigned the same number as their complementary nucleotides on the non-target strand. Potential DNA-protein interactions are indicated with a dashed line and atomic distance. Amino acid position 884 is represented by a Coulombic electrostatic surface potential map and encircled with a dashed line (red, negative; blue, positive; white, non-polar). NTS, non-target strand; TS, target strand; PAM, protospacer adjacent motif. See also Figures S1–S3 and Table S1.

Prior studies demonstrated that the three WED-domain mutations, E843K, E884G, and K908R, in iGeoCas9 had the biggest impact on promoting genome-editing activity, but the mechanism was unclear.^9^ The ~100-amino-acid WED domain comprises 4 α helices and 2 β sheets that recognize the repeat:anti-repeat region of the sgRNA and the dsDNA located upstream of the target region (Figure 2A). The iGeoCas9 WED-domain mutations (E843K, E884G, and K908R) may alter contacts with the phosphate backbone of the target adjacent dsDNA. Indeed, the positively charged lysine (K) at position 843 in iGeoCas9 is situated to establish a new electrostatic interaction with the negatively charged phosphate backbone of TS DNA (Figure 2B). Mutation K908R in iGeoCas9, located in the minor groove adjacent to the NTS, potentially enhances DNA binding electrostatics (Figure 2B). Together, mutations E843K and K908R in iGeoCas9 may augment DNA strand separation and R-loop formation required for DNA cleavage. Although not in contact with nucleic acid in our experimental structure, the glutamate 884 to glycine mutation (E884G) in iGeoCas9 may further enhance nucleic acid interactions through altering the electrostatics (Figure 2B). Based on these structural observations, we hypothesize the three WED-domain mutations in iGeoCas9 favor DNA strand separation prior to cleavage. This motivated us to biochemically investigate how these mutations affect each individual step of the Cas9 catalytic pathway (Figure 1B).

### iGeoCas9-catalyzed DNA cleavage has a relaxed PAM requirement

**Figure 3.**
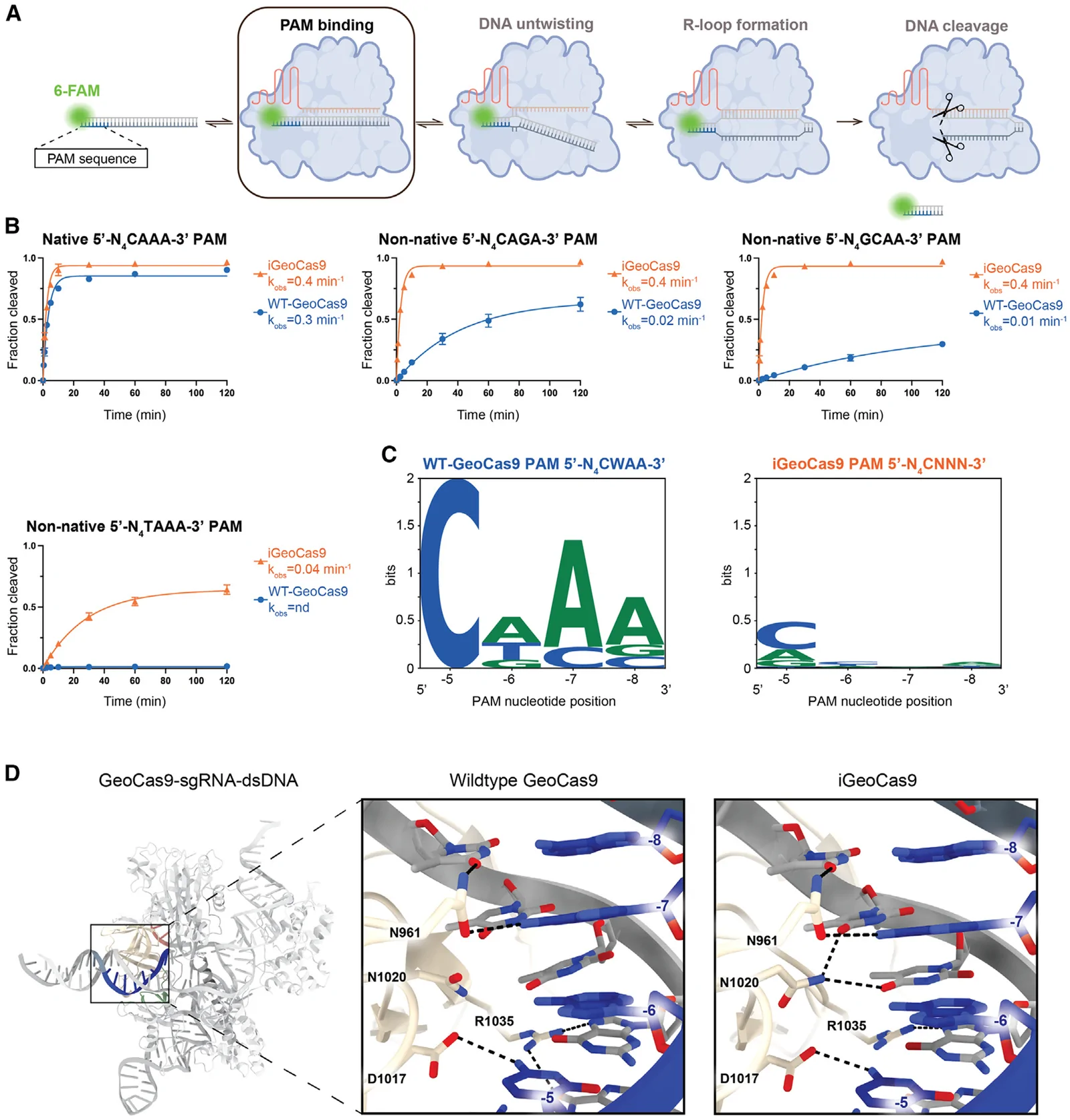
iGeoCas9 demonstrates enhanced activity targeting wild-type GeoCas9 non-native PAMs (A) Schematic of the Cas9 cleavage reaction using 60 nucleotide (nt) 5′ 6-FAM labeled double-stranded (ds) DNA substrates with different PAM sequences. Nucleic acid is colored as in Figure 1D. (B) *In vitro* dsDNA cleavage activity of wild-type (WT-)GeoCas9 and iGeoCas9 determined by denaturing PAGE (*n* = 3, data are represented as mean ± SD). PAM contained in each substrate indicated above the graph. Fractions were collected at 0 s, 30 s, 1 min, 2.5 min, 5 min, 10 min, 30 min, 1 h, and 2 h. Fraction “0” is represented by the substrate only. The k_obs_ for each Cas9 are listed in the sample legend. See also Figure S4A. (C) Logo for sequences depleted from the PAM library by wild-type (WT-)GeoCas9 (left) and iGeoCas9 (right). Consensus PAM sequence located above the logo. PAM position on x axis and is numbered in the 5′ to 3′ direction in descending order from −5 to −8. C and T, blue; and A and G, green. (D) PAM nucleobase-interacting amino acids (N961, N1020, D1017, and R1035) of wild-type GeoCas9 and iGeoCas9. PAM sequence position indicated adjacent to the nucleotide. H-bond prediction was performed in ChimeraX v1.6.1^11^ with a distance tolerance of 0.400Å and angle tolerance of 20°. Alternate rotamer conformations were observed for N1020 and R1035 in wild-type GeoCas9 and iGeoCas9. See also Figure S4A.

Cas9 interacts with a protospacer-adjacent motif before it can proceed with DNA TS recognition and cleavage, the necessary precursor to genome editing.^10^ To determine how the mutations in iGeoCas9 promote genome editing, we first investigated their effect on PAM sequence recognition. The PAM was previously determined as 5′-N_4_CRAA-3′ (where R = A/G, N = A/T/C/G) for wild-type GeoCas9.^7^ Using a 6-carboxyfluorescein (6-FAM)-labeled 60-base-pair DNA substrate, we observed that wild-type GeoCas9 and iGeoCas9 have similar DNA cleavage activities against the native 5′-N_4_CAAA PAM (Figures 3A and 3B). When dsDNA bearing different PAM sequences were employed as substrates, we found that iGeoCas9 has a much wider tolerance for non-native PAMs (5′-N_4_CAGA-3′, 5′- N_4_GCAA-3′, and 5′- N_4_TAAA-3′) compared with wild-type GeoCas9 (Figures 3B and S4A). These observations show that iGeoCas9 has acquired the ability to accommodate more diverse PAM sequences, allowing it to target a broader range of substrate sequences.

Next, we used a PAM depletion assay to compare the behavior of wild-type GeoCas9 and iGeoCas9 more comprehensively.^12^ In this assay, Cas9 targets a population of PAMs, identifying well-represented nucleotide combinations. PAMs modestly recognized by Cas9 may not be represented. Purified guide RNA-Cas9 RNP complexes were incubated with a plasmid library containing a PAM with four randomized nucleotides at positions −5 to −8 (5′-TTTTN_4_-3′). Successful PAM recognition depletes those sequences from the library relative to a non-targeting guide RNP control. Next-generation sequencing (NGS) revealed wild-type GeoCas9 was constricted to a consensus sequence of 5′-N_4_CWAA-3′ (where W = A/T), consistent with our prior report.^7^ PAM depletion results showed that iGeoCas9 uses a relaxed PAM consensus sequence of 5′-N_4_CNNN-3′ (Figure 3C). Although PAM specificity can be altered for different Cas proteins when mutations are introduced to the PAM-interacting (PI) domain,^13,14^ iGeoCas9 does not contain mutations in the PI domain (Figure 3D). PAM expansion is likely driven by the DNA backbone interactions in the WED domain.^15^ Together, these observations suggest that downstream steps in DNA unwinding or catalysis contribute to the expanded PAM compatibility of iGeoCas9.

### iGeoCas9 has improved DNA-melting kinetics

**Figure 4.**
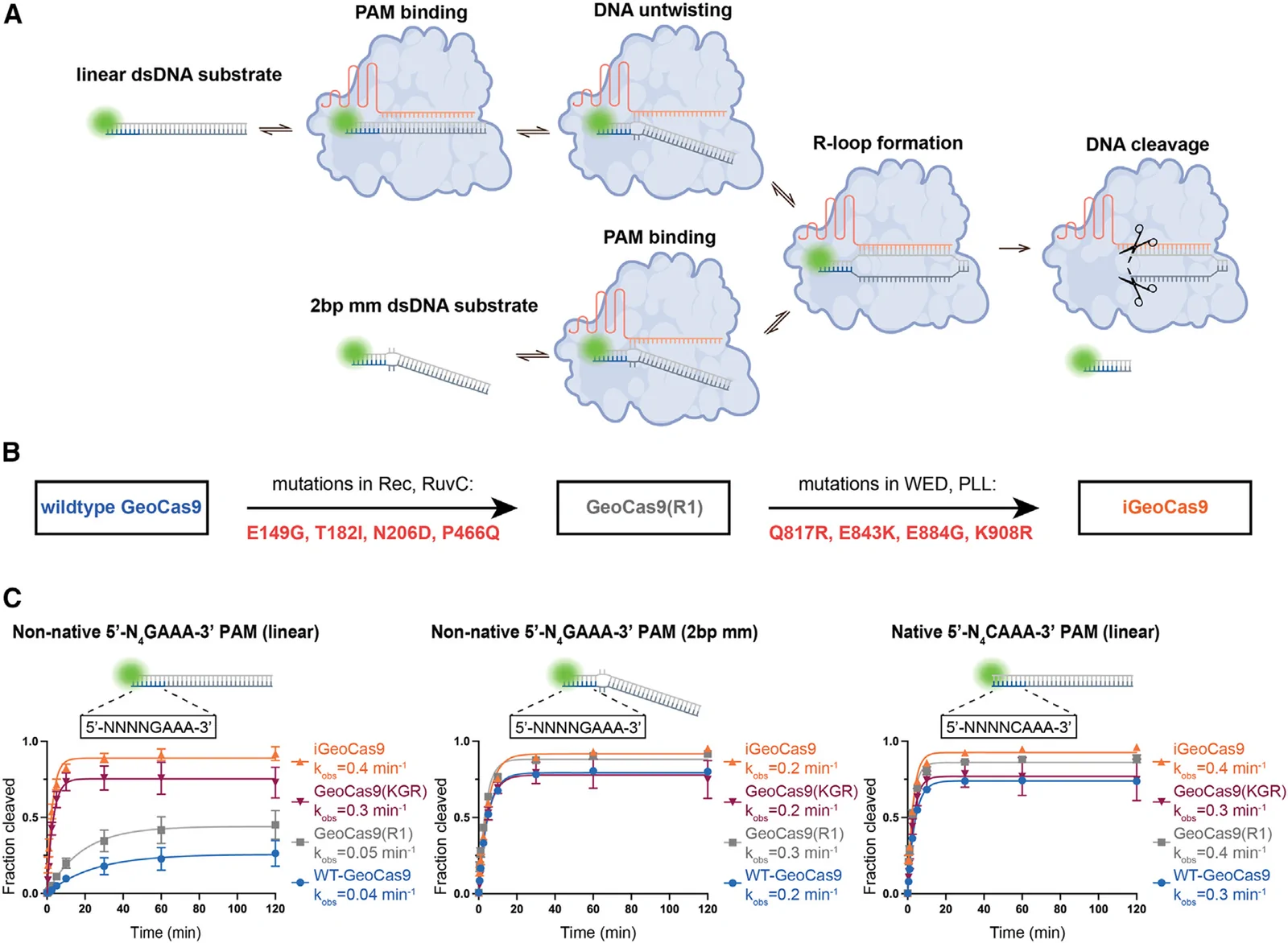
Thermodynamically unstable substrate mimics WED-domain mutation effects on DNA melting (A) Schematic of the Cas9 cleavage pathway for linear dsDNA substrates versus two base pair mismatch (2 bp mm) dsDNA substrate. The 60 nt DNA substrate is 5′ 6-FAM labeled (green). Nucleic acid is colored as in Figure 1D. (B) The order in which mutations were introduced to create GeoCas9(R1) and iGeoCas9. Mutations are listed below the arrow, and domains in which they are located are above the arrow. WED, wedge; PLL, phosphate lock loop. (C) *In vitro* dsDNA cleavage activity of wild-type (WT-)GeoCas9, iGeoCas9, GeoCas9(R1), and GeoCas9(KGR) determined by denaturing PAGE (*n* = 3, data are represented as mean ± SD). Substrate and PAM sequences are indicated above the graph. Fractions were collected at 0 s, 30 s, 1 min, 2.5 min, 5 min, 10 min, 30 min, 1 h, and 2 h. Fraction “0” is represented by the substrate only. The k_obs_ for each Cas9 are listed in the sample legend. See also Figure S4B.

The cryo-EM structures of wild-type GeoCas9 and iGeoCas9 suggest that mutations K908R and E843K in iGeoCas9 may enhance DNA backbone interactions on the NTS and TS of the recognition sequence. To test whether the effect of these mutations is to improve DNA unwinding upon Cas9-guide target binding, we used DNA substrates containing either perfectly matched (linear) or mismatched (mm) base pairs immediately adjacent to the PAM sequence (Figure 4A). Previous studies demonstrated that type II-C Cas9s have improved DNA unwinding and DNA cleavage kinetics with a thermodynamically destabilized DNA substrate containing two base pair mismatches adjacent to the PAM.^16^ These mismatches assist in the initial disruption of the dsDNA helix, or DNA melting, that accompanies R-loop formation (Figure 4A). To test whether the WED-domain mutations change the ability of GeoCas9 to recognize and cleave DNA target sequences that have a non-native PAM, we designed a 60-bp DNA substrate bearing a PAM sequence, 5′-N_4_GAAA-3′, that is disfavored by wild-type GeoCas9. To further explore the effect of WED-domain mutations, activities of wild-type GeoCas9 and iGeoCas9 were compared with an intermediate mutant from iGeoCas9’s laboratory evolutionary lineage, GeoCas9(R1),^9^ that does not contain the WED-domain mutations, and another variant that only contains the WED-domain mutations, GeoCas9(KGR) (Figures 4B and S4B). Under substrate-limiting (single-turnover) conditions with a linear substrate, initial rates for both the wild-type GeoCas9 and GeoCas9(R1) were similar (0.04 versus 0.05 min^−1^), suggesting the mutations in GeoCas9(R1) had little effect on catalysis. By contrast, iGeoCas9 and GeoCas9(KGR) exhibited >5-fold-faster DNA cleavage kinetics (0.4 and 0.3 min^−1^) compared with the wild-type or GeoCas9(R1) enzymes, underscoring the importance of WED-domain mutations in promoting enzyme activity (Figures 4C and S4B). Next, to test if the WED mutations affect DNA target sequence recognition, we redesigned the dsDNA substrate to include mismatches in the first two base pairs of the target region adjacent to a suboptimal 5′-N_4_GAAA-3′ PAM. Under single-turnover conditions, all three enzymes had similar initial rates for the cleavage of this substrate (0.2–0.3 min^−1^), indicating a 5-fold increase in rate for both the wild-type GeoCas9 and GeoCas9(R1) enzymes, leading to activity levels similar to iGeoCas9 and GeoCas9(KGR) (Figures 4C and S4B). This observation suggested that destabilization of the DNA double strands where target DNA and guide RNA base pairing begins compensates for the absence of iGeoCas9’s WED-domain mutations. When tested with a linear substrate containing the native 5′-N_4_CAAA-3′ PAM, all three enzymes had similar cleavage kinetics (wild-type GeoCas9, 0.3 min^−1^; iGeoCas9, 0.4 min^−1^; GeoCas9(R1), 0.4 min^−1^; GeoCas9(KGR), 0.3 min^−1^) (Figures 4C and S4B). Together, these data show that enzymes lacking the WED-domain mutations, including wild-type GeoCas9 and GeoCas9(R1), require substrate destabilization by base pair mismatches to overcome the presence of a suboptimal PAM. These findings imply that the WED-domain mutations supersede the role of the PAM in DNA target recognition by perhaps enhancing GeoCas9’s DNA untwisting capability.

### Faster R-loop formation by iGeoCas9 compensates for magnesium-restricted conditions

**Figure 5.**
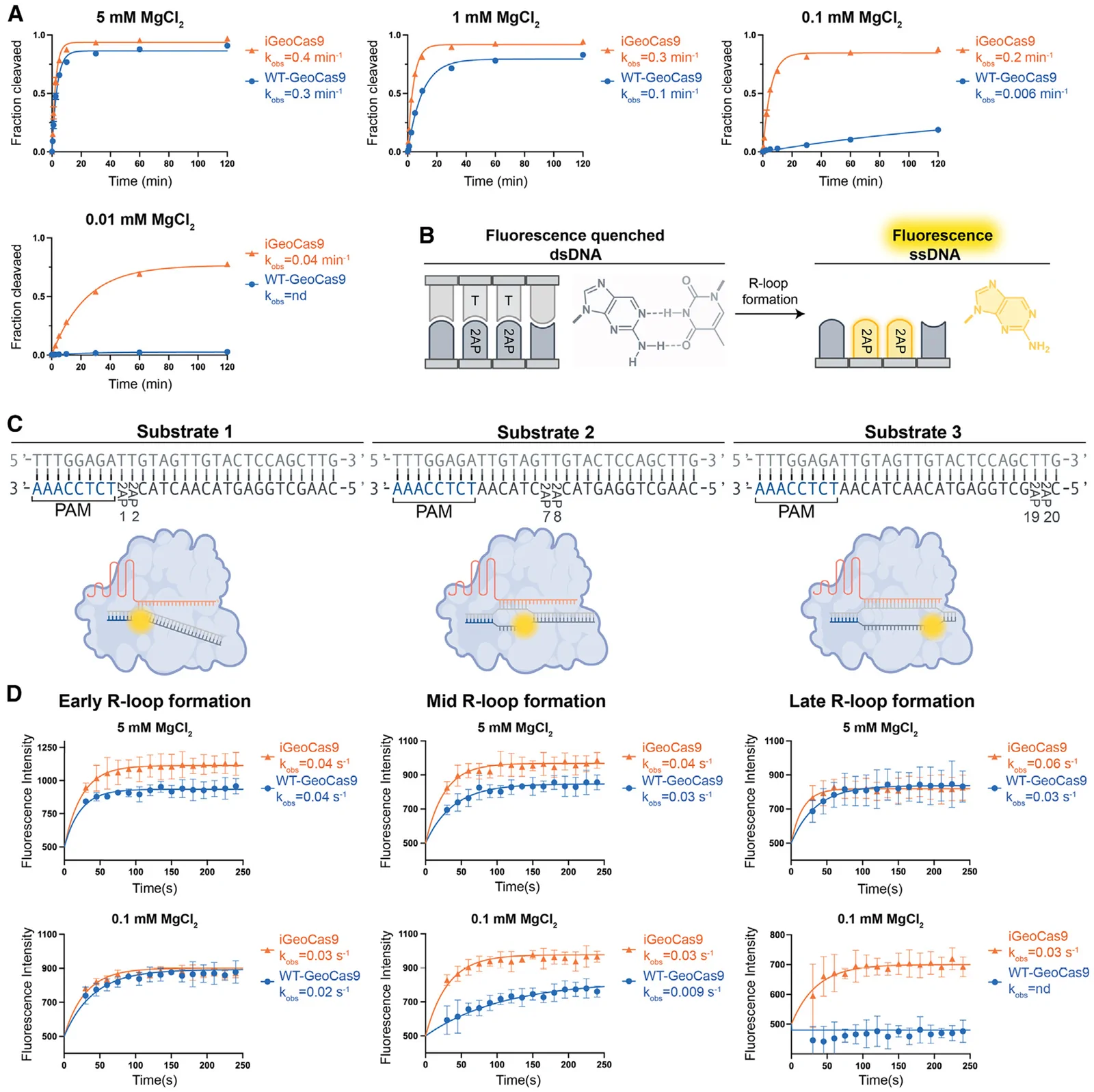
Reduced concentration of MgCl_2_ impacts wild-type GeoCas9 R-loop formation (A) *In vitro* dsDNA cleavage activity of wild-type (WT-)GeoCas9 and iGeoCas9, determined by denaturing PAGE (*n* = 3, data are represented as mean ± SD). MgCl_2_ concentration indicated above the graph. Fractions were collected at 0 s, 30 s, 1 min, 2.5 min, 5 min, 10 min, 30 min, 1 h, and 2 h. Fraction “0” is represented by the substrate only. The rate constants k_obs_ are listed in the sample legend. See also Figure S4C. (B) Cartoon and chemical structure of 2-aminopurine (2AP) in the quenched and fluorescent states. Fluorescence indicated with yellow. T, thymine; dsDNA, double-stranded DNA; ssDNA, single-stranded DNA. (C) Diagram of substrates (partial sequence) indicating positions of 2AP nucleotides measuring 5′ from the PAM (top). Drawing of the different stages of GeoCas9 R-loop formation with the three different substrates (bottom). Fluorescent 2APs are indicated in yellow. (D) 2AP fluorescence assays comparing catalytically inactivated wild-type (WT-)GeoCas9 and iGeoCas9 R-loop kinetics (*n* = 3, data are represented as the mean ± SD). Substrate 1, early R-loop formation. Substrate 2, mid R-loop formation. Substrate 3, late R-loop formation. MgCl_2_ concentrations indicated above the graph. The rate constant k_obs_ are listed in the sample legend. See also Figure S4C.

Wild-type GeoCas9 is ineffective for genome editing in mammalian cells, even when targeting genomic loci with optimal PAM sequences. By contrast, iGeoCas9 induces genome edits with >100-fold-higher efficiency despite similar DNA cleavage kinetics compared with wild-type GeoCas9 using optimal substrates *in vitro*. One important difference between bacterial and mammalian cells is the availability of free magnesium ions, which can affect the formation of protein-nucleic acid complexes and Cas effector specificity.^17,18^ Bacterial cells and our biochemical assays have free magnesium concentrations of >1 mM,^19^ whereas mammalian cells contain much lower levels of free magnesium ion of 0.1–1 mM.^20,21^ To test the effect of magnesium ion concentration on enzyme activity, we determined DNA cleavage rates under single-turnover conditions for wild-type GeoCas9 and iGeoCas9 at 5–0.01 mM magnesium chloride (MgCl_2_) concentrations. Using a 6-FAM-labeled substrate with a native 5′-N_4_CAAA-3′ PAM, we found that wildtype GeoCas9 and iGeoCas9 had similar single-turnover cleavage kinetics at 5 mM MgCl_2_ concentration. However, a titration of magnesium concentrations from 1 to 0.1 mM resulted in a ~17-fold decrease in wild-type GeoCas9-catalyzed DNA cleavage. Surprisingly, with a MgCl_2_ concentration as low as 0.01 mM, iGeoCas9 remains active with a k_obs_ of 0.04 min^−1^. Under the same conditions, wild-type GeoCas9 activity dropped to a k_obs_ below the detection limit (Figures 5A and S4C). These data suggest that iGeoCas9 is less dependent on magnesium, enabling it to maintain high activity in environments where free magnesium is less available, such as mammalian cells.

The significant reduction of wild-type GeoCas9 activity at the low magnesium concentration found in human cells suggested a potential explanation for its poor genome-editing activity in this cell type. Furthermore, we wondered whether the mutations in iGeoCas9, which enable activity at very low magnesium ion concentrations *in vitro*, might have altered the rate-determining step of DNA cleavage. Since guide RNA strand invasion to form an R-loop with the DNA target sequence was previously determined to be the rate-limiting step for SpyCas9,^18,22^ we tested whether there is a difference in the ability of wild-type GeoCas9 versus iGeoCas9 to form an R-loop under different magnesium ion conditions.

To investigate R-loop formation kinetics, we introduced 2-aminopurine (2AP) fluorescent nucleotides^22,23^ into the NTS of the dsDNA substrate containing an optimal PAM 5′-N_4_-CAAA-3′ sequence. In the dsDNA helix, 2AP fluorescence is quenched through base-stacking interactions with neighboring nucleotides. The unwinding and destacking of dsDNA by Cas9 results in 2AP fluorescence (Figure 5B).^24^ To determine R-loop formation kinetics at different stages, dsDNA substrates were designed to contain tandem 2AP markers located at varying distances from the PAM. Three substrates with 2APs at positions 1&2, 7&8, or 19&20 of the target region were used to determine the early, mid, and late stages of R-loop formation kinetics, respectively (Figure 5C). Consistent with prior measurements, no major differences in kinetics were observed between wild-type GeoCas9 and iGeoCas9 when testing the three substrates in the presence of 5 mM MgCl_2_ (Figure 5D). Interestingly, when the MgCl_2_ concentration was reduced to 0.1 mM, reactions using substrate 1 with 2APs at positions 1&2 showed similar early R-loop formation kinetics for both wild-type GeoCas9 and iGeoCas9, suggesting there is no major difference in the initial DNA interrogation step when using an optimal PAM 5′-N_4_CAAA-3′ (Figure 5D). However, a 3.3-fold difference was observed for the kinetics of mid-R-loop formation (2AP at positions 7&8) when comparing wild-type GeoCas9 and iGeoCas9. More surprisingly, the kinetic analyses of late R-loop formation (2AP at positions 19&20) established that wild-type GeoCas9 is exceptionally slow at dsDNA unwinding under MgCl_2_-restricted conditions, while iGeoCas9 maintained fast kinetics for complete R-loop formation with a kinetic constant (0.03 s^−1^), the same as early R-loop (0.03 s^−1^) (Figure 5D).

R-loop kinetic measurements show that in the presence of high MgCl_2_ concentrations (e.g., 5 mM), early-, mid-, and late-R-loop formation were similar for wild-type GeoCas9 and iGeoCas9. This suggests that under these conditions, R-loop formation is not the rate-determining step. Under conditions of low MgCl_2_ concentration (e.g., 0.1 mM), mid- and late-R-loop formation by wild-type GeoCas9 was slow, suggesting that R-loop formation has become rate-determining. By contrast, iGeoCas9 quickly progressed through all stages of R-loop formation at low MgCl_2_. Together, these results establish that the mutations in iGeoCas9 substantially improve DNA unwinding capability in the mid and late stages of R-loop formation, enabling sustained enzyme activity under low magnesium ion conditions.

### Mutations accelerating R-loop formation are transferable to another genome editor

**Figure 6.**
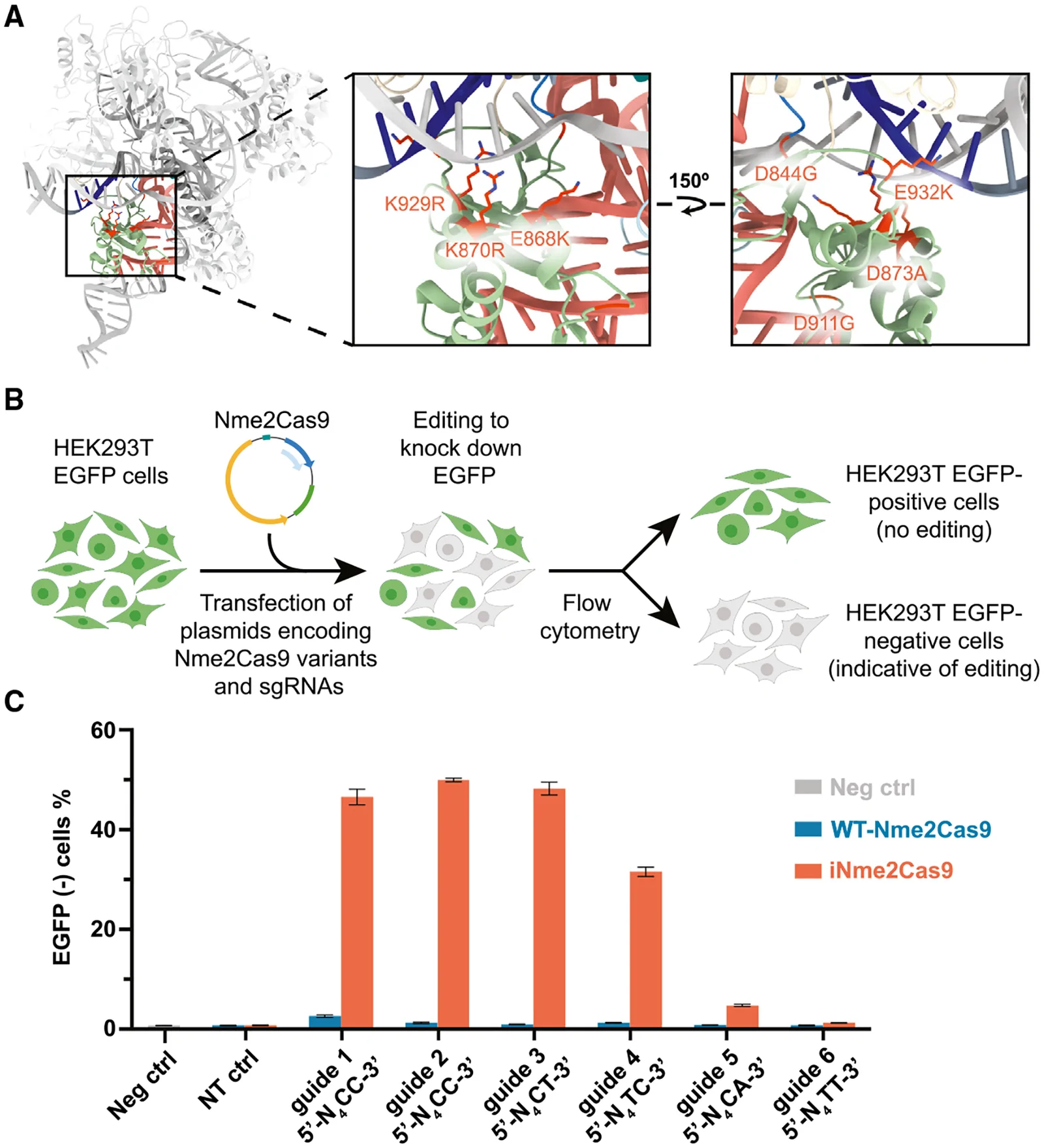
WED-domain mutations greatly enhance genome-editing activities of Nme2Cas9 (A) Model of Nme2Cas9 (PDB: 6JE3)^26^ in which all seven rationally engineered mutations are represented in the model as red sticks. Rotamers were chosen to demonstrate potential DNA interactions. (B) Workflow for EGFP-knockdown assay in HEK293T cells. Successful editing indicated by a loss of EGFP signal. (C) HEK293T cell editing by wild-type (WT-) Nme2Cas9 and iNme2Cas9 and 6 different guides (*n* = 4, data are represented as the mean ± SD). Neg, no treatment control; NT, non-targeting guide control. See also Figures S5 and S6.

Having established that iGeoCas9 WED-domain mutations are likely to promote genome-editing efficiency through expedited R-loop formation and reduced PAM specificity, we wondered whether these biochemical insights might enable rational engineering of other Cas9 proteins. Nme2Cas9,^25,26^ with ~38% sequence identity to GeoCas9, was selected as a candidate protein for engineering. Similar to iGeoCas9, WED-domain mutations were introduced into Nme2Cas9 to generate two new, improved versions of Nme2Cas9. Nme2Cas9(v1) contains E868K, K870R, and K929R mutations to allow for potential new DNA TS interactions, and D873A and D911G mutations that could mimic the E884G mutation in iGeoCas9 (Figures 6A and S5A). Two additional mutations, E932K and D844G, expected to interact with the non-target DNA strand and alter protein charge, respectively, were further introduced to generate iNme2Cas9 (Figures 6A and S5B). The genome-editing activities of wild-type Nme2Cas9, Nme2Cas9(v1), and iNme2Cas9 were evaluated using an enhanced green fluorescent protein (EGFP) knockdown assay in HEK293T cells. Plasmids encoding Nme2Cas9 proteins together with sgRNAs were transfected to HEK293T cells, which were analyzed by flow cytometry to give editing efficiencies (Figure 6B). Six guide RNAs, guides 1–6, were designed to target the EGFP transgene using various PAM sequences. 5′-N_4_CC-3′ was previously identified as the optimal PAM sequence for wild-type Nme2Cas9.^25^ Guides 1 and 2 were designed to use the native 5′-N_4_CC-3′ PAM, and the corresponding editing tests to disrupt EGFP revealed substantial improvement in the editing efficiency from wild type to Nme2Cas9(v1) and iNme2Cas9 (Figures 6C and S5C). Based on our demonstration that the WED-domain mutations in iGeo-Cas9 led to relaxed PAM recognition, we expected to see an expansion in PAM sequences with our engineered Nme2Cas9 variants. Therefore, guides 3–6, targeting the EGFP gene with non-native PAMs, 5′-N_4_CT-3′, 5′-N_4_TC-3′, 5′-N_4_CA-3′, and 5′-N_4_TT-3′, respectively, were also included in our editing test. As expected, our engineered iNme2Cas9 was able to give up to 50% EGFP-knockdown efficiency with guides 3–6, while the wild-type Nme2Cas9 barely induced any editing activity (Figure 6C). Overall, our iNme2Cas9 showed up to >100-fold improvement in genome-editing activity compared with the wild-type enzyme.

Nme2Cas9 has been previously engineered using a phage-assisted continuous evolution (PACE) system by the Liu lab.^27^ We were thus interested in comparing our engineered iNme2Cas9 to the reported variants. We selected two nuclease-reactivated mutants from the study, Nme2Cas9(C-NR) and Nme2Cas9(T-NR), which recognize C-based and T-based PAM sequences, respectively. Using the same EGFP-knockdown assay under dose-limiting conditions, we were delighted to observe that our rationally designed iNme2Cas9 outperformed the previously engineered mutants using five guide RNAs and single-pyrimidine PAMs (Figure S5D). These results have further reinforced that the WED-domain mutations are responsible for accelerating R-loop formation under magnesium-restricted conditions, typical of mammalian cell environments, which, however, may not be preferentially selected out using bacterial-based evolution systems like PACE. Taking all these together, we believe our rational engineering of Nme2Cas9 supports that optimizing Cas9 WED domain can lead to robust genome editors with expanded PAM compatibility and improved editing efficiency.

To further compare these wild-type and engineered type II-C Cas9s (i.e., wild-type, iNme2Cas9, and iGeoCas9) with the state-of-the-art genome editor, type II-A SpyCas9, we performed genome-editing tests targeting endogenous sites (e.g., *EMX1* and *AAVS1*) with HEK293T cells, using a consensus PAM of 5′-NGGNCTAA-3′ (Figure S6A). Overall, we observed comparable editing efficiency between iGeoCas9 and SpyCas9 across four different target sites, while iNme2Cas9 or Nme2Cas9(C-NR) were slightly less effective but still showed improved editing over the wild-type Nme2Cas9. Further analysis revealed that the engineered type II-C Cas9s had barely detectable promiscuous editing events at off-target sites predicted by Cas-OFFinder (v3.0.0b3)^28^ (Figure S6B). Overall, this supports the importance of the WED domain in improvement of genome editors with iGeoCas9 having the greatest activity of these type II-C editors, reaching activity levels similar to SpyCas9 while preserving their editing fidelity.

## DISCUSSION

CRISPR-Cas9-based technology has diverse applications across the life sciences.^5,29^ In particular, Cas9 has the therapeutic potential to treat a wide range of genetic diseases.^30,31^ To expand its utility, the basic functions of CRISPR-Cas9 have been extended using protein engineering.^32^ For example, engineered SpyCas9 enzymes can accommodate a range of non-NGG PAM sequences, enabling them to target a wider range of genomic sites.^13,14,33^ Cas9 proteins have also been rationally optimized to have higher fidelity of DNA cleavage, leading to reduced off-target editing.^34–36^ However, protein engineering has demonstrated little success in improving Cas9’s basic genome-editing activities.^37^ This may be because we lack a proper understanding of the essential relationship between Cas9 structure and its genome-editing activities. A separate study demonstrated that engineering a thermostable type II-C GeoCas9 can produce a robust genome editor, iGeoCas9, with substantially improved editing efficiency in cultured mammalian cells and animal tissues.^9^ Biochemical and structural comparison of wild-type GeoCas9 versus iGeoCas9 as reported here provides insights into the structure-function relationship of Cas9 and establishes general principles for engineering Cas9’s genome-editing activities.

Our work reveals the pivotal and unanticipated role that the WED domain plays in regulating type II-C Cas9’s genome-editing activities. We found that WED-domain mutations in iGeoCas9 establish new interactions with the DNA TS and NTS backbones in the PAM region of the dsDNA target. These interactions enhance R-loop formation, the DNA interrogation step that involves unwinding the target dsDNA sequence to enable RNA-DNA duplex formation. R-loop formation is the rate-limiting step in Cas9-catalyzed DNA cleavage,^17,22^ and slow R-loop formation kinetics likely limits GeoCas9’s editing activities in mammalian cells. We found that the evolved editor, iGeoCas9, was able to overcome the slow R-loop kinetics observed with wild-type GeoCas9 in low magnesium environments. This effect could be due to the observed increased interactions between the WED domain and target dsDNA, leading to a dramatically accelerated R-loop formation process. This communication between the WED domain and the PAM distal end of the R-loop could be accomplished through conformational changes, similar to SpyCas9.^38^ This makes R-loop formation no longer the rate-limiting step in catalysis and also imposes a much less stringent requirement on PAM recognition for target binding.

Having evolved as powerful immune defense machinery in prokaryotic organisms, heterologous biological environments may alter or restrict the targeted endonucleolytic function of CRISPR-Cas enzymes. A better understanding of the mechanisms and constraints of CRISPR enzymes, followed by the development of general engineering strategies to attenuate these constraints, can help establish robust and effective genome-editing tools. Our study illustrates that enhanced binding between the Cas9-guide RNA enzymatic complex and the target dsDNA corresponds to increased genome-editing efficiency. In particular, the three WED-domain mutations in iGeoCas9 create new DNA TS and NTS backbone interactions and alter the electrostatic environment of the protein-DNA interface, accelerating R-loop formation under magnesium-restricted conditions. Similar engineering of the related Nme2Cas9 protein generated a mutant editor showing remarkable improvement for mammalian cell genome editing. Several groups have reported an engineering strategy that involves introducing cationic residues to enhance target DNA binding, thereby improving the function of CRISPR-Cas or relevant proteins.^39–41^ However, this strategy requires intensive mutational screening at residues surrounding target DNA or even arginine scanning throughout the entire protein. Our findings highlight the WED domain as a previously unappreciated regulator of the mechanisms that drive Cas9’s genome-editing behaviors, particularly in mammalian cells with low free magnesium content. The WED domain, functioning as a bridge between the sgRNA (or CRISPR RNA [crRNA]) scaffold and the upstream dsDNA region of the target DNA, can be identified in CRISPR-Cas9, Cas12, and their relevant proteins, including IscB,^42^ TnpB,^43^ and Fanzor^44^ proteins. Despite different structural organizations of the WED domain among the diverse systems, we assume that engineering of the WED domain (or other related regions with similar structural functions, e.g., PI domain) can possibly be applied to these systems based on the simple principle to enhance the binding to target DNA. Preliminary findings with the state-of-the-art genome editor, type II-A SpyCas9, albeit featuring different C-terminal domain organization (WED + PI) compared with type II-C Cas9s, show that it can also be potentially engineered with improved editing efficiency (Figure S6C). Therefore, we believe focusing engineering efforts on the dsDNA-binding regions of CRISPR-Cas proteins can be a streamlined approach that holds promise for the development of more effective genome-editing systems and would lead to more robust CRISPR-based therapeutics.

### Limitations of the study

Some mechanistic details remain to be uncovered about wild type and iGeoCas9 such as if magnesium ions are bound in any specific region in the protein structures and how Mg^2+^ contributes to R-loop formation. As Mg^2+^ could only be reliably assigned with structure resolution below 3Å,45 the cryo-EM methods in this study were not optimal for identifying Mg^2+^ coordination sites. Revisiting the X-ray ternary structure of SpyCas9 (PDB: 4UN3),^10^ multiple magnesium-binding sites were identified, including two involved in the duplex of target DNA strand and sgRNA spacer. It would be reasonable to hypothesize GeoCas9 may occupy magnesium ions in a similar scenario, but this will require further experimental validation. An additional limitation to the study is that a ternary structure of a genome-editor enzyme is required to rationally engineer its WED domain for activity improvement. This may limit the scope of Cas enzymes that can be rationally designed to those that have structural data. Nevertheless, fast-advancing computational tools for protein structural prediction, as exemplified by AlphaFold-latest^46^ that has been used to predict the ternary structure of Casλ,^47^ can be possibly used to assist the rational engineering of other CRISPR genome editors.

## STAR★METHODS

### RESOURCE AVAILABILITY

#### Lead contact

Further information and requests for resources and reagents should be directed to and will be fulfilled by the lead contact, Jennifer A. Doudna (doudna@berkeley.edu).

#### Materials availability

Plasmids generated in this study will be deposited to Addgene upon publication. Addgene IDs are available in the key resources table. This study did not generate new unique reagents.

#### Data and code availability

- PAM depletion, on-target editor comparison, and off-target analysis sequencing data are publicly available as of the date of publication. The project number is listed in the key resources table. Original denaturing PAGE image files are available from lead contact upon request. Raw 2D electron microscopy data is available from lead contact upon request.
- All original code has been deposited to Zenodo and is publicly available as of the date of publication. DOI is listed in the key resources table.
- Any additional information required to reanalyze the data reported in this paper is available from the lead contact upon request.

### EXPERIMENTAL MODEL AND STUDY PARTICIPANT DETAILS

#### Mammalian cell culture

Genome-editing experiments were performed in HEK293T EGFP reporter cells and editor comparison of on-target and off-target activity were performed in HEK293T cells. Both cell lines were purchased from the University of Berkeley California Cell Culture Facility and were cultured at 37°C with DMEM media (Corning), 10% fetal bovine serum, and 100 U/mL penicillin-streptomycin (Gibco).

### METHOD DETAILS

#### Oligonucleotides

Oligonucleotides used in this study are listed in Table S2.

#### Plasmids and cloning

Wildtype GeoCas9 and iGeoCas9 plasmids without NLS tags were prepared using plasmids 2NLS-GeoCas9(WT)-2NLS and NLS-GeoCas9(R1W1)-2NLS and cloned by PCR of the plasmid backbone and desired insert. Amplified fragments contained a 24 bp overlapping region and were assembled using Gibson assembly to create plasmids His-CL7 MBP WTGeoCas9 and His-CL7 MBP iGeoCas9(R1W1).

Nuclease domains were inactivated by PCR with primers containing the desired inactivating sequences (D8A and H582A). PCR fragments contained a 17–21bp overlap and were assembled using Gibson assembly to create plasmids pARE110 His-CL7 MBP dWTGeoCas9 and pARE112 His-CL7 MBP diGeoCas9(dR1W1).

#### GeoCas9 protein expression and purification

Protein expression and purification were performed previously as described with some modifications.^7^ Briefly, *E. coli* BL21 DE3 cells (NEB) were transformed with a Cas9 expression plasmid (pARE110_His-CL7_MBP_dWTGeoCas9, pARE112_His-CL7_MBP_ diGeoCas9(dR1W1), NLS-GeoCas9(R1W1)-2NLS, 2NLS-GeoCas9(WT)-2NLS, NLS-GeoCas9(R1)-2NLS, 2NLS-GeoCas9(KGR)-2NLS, His-CL7 MBP WTGeoCas9 and His-CL7 MBP iGeoCas9(R1W1) in 2xYT medium supplemented with 100 ug/ml ampicillin and grown to an OD between 0.6 to 0.8. Cultures were cooled on ice for 30 min then supplemented with 0.5 mM isopropyl b-D-thiogalactoside (IPTG) and grown overnight at 16°C. Cells were harvested by centrifugation, gently resuspended in lysis buffer (50 mM Tris-HCl, pH 7.5, 20 mM imidazole, 0.5 mM tris(2-carboxyethyl)phosphine (TCEP), 500 mM NaCl, 1 mM PMSF), and lysed by sonication. Clarified lysate was incubated with Ni-NTA resin (Qiagen) pre-equilibrated with wash buffer (50 mM Tris-HCl, pH 7.5, 20 mM imidazole, 0.5 mM TCEP, 500 mM NaCl) for 1 hour. Protein bound resin was washed 3 times and eluted with Ni-NTA elution buffer (50 mM Tris-HCl, pH 7.5, 500 mM imidazole, 0.5 mM TCEP, 500 mM NaCl). Pierce Human Rhinovirus 3C Protease (Thermo Fisher Scientific) was added to the elution for cleavage and dialyzed overnight in a 10,000 MWCO dialysis cassette (Thermo Fisher Scientific) with dialysis buffer (20 mM HEPES pH 7.5, 150 mM KCl, 10% glycerol, and 1 mM TCEP) overnight at 4°C. Proteins were filtered with a 0.22 uM filter unit (Millex GP), injected into a pre-equilibrated MBPTrap HP column (Cytiva) and washed buffer A (20 mM HEPES pH 7.5, 150 mM KCl, 10% glycerol, and 1 mM TCEP) until 260 and 280 absorbance readings reached UV baseline. Proteins were eluted with a linear gradient of KCl concentrated with a 30,000 MWCO concentrator (Millipore Sigma) and loaded onto a Superdex 200 Increase 10/300 GL (Cytiva) and purified using gel filtration buffer (20 mM HEPES, pH 7.5, 150 mM KCl, 10% glycerol, 1 mM TCEP). Peak fractions containing Cas9 were quantified using a NanoDrop 8000 Spectrophotometer (Thermo Scientific) and flash frozen and stored at −80°C.

#### Nucleic acid preparation

DNA substrates and sgRNA was ordered from Integrated DNA Technologies (IDT). Substrates were purified in house using a 12% urea-PAGE gel. The band containing the substrate was excised, gentle ground, and incubated in 1:10 volume of 3M sodium acetate overnight at 4°C. Samples were 0.22 um vacuum filtered and concentrated with a 3 kDa MWCO centrifugal filter (MilliporeSigma). Samples were ethanol precipitated with greater than 2.5 times the volume of 100% ethanol at −80°C for 2 hours. Samples were pelleted using centrifugation, washed twice with 80% ethanol, and resuspended in molecular grade water. Samples were stored at −80°C for later use.

#### Cryo-EM ternary complex formation

Target DNA (doARE791 and doARE792) was annealed in water by heating to 94°C for 2 minutes and slowly cooling to room temperature on a ProFlex PCR system. sgRNA (roARE062) was annealed in 1 × annealing buffer (10 mM Tris, pH 7.5, 20 mM KCl, 1.5 mM MgCl_2_) by heating at 80°C for 2 minutes then placing it on ice. Nuclease inactivated wildtype GeoCas9 or iGeoCas9 was incubated with sgRNA (1:1.3) in 1 × cleavage buffer (20 mM Tris-HCl, pH7.5, 100 mM KCl, 5 mM MgCl_2,_ 1 mM TCEP, 5% (w/v) glycerol) at room temperature for 30 min followed by 5 min at 37°C for RNP formation. Target dsDNA was added to RNP (1:1) and incubated at 37°C for 2 hours for ternary complex formation (final concentration 10 μM). Ternary complex was loaded onto a Superdex 200 Increase 10/300 GL (Cytiva) and purified using CryoEM buffer (20 mM Tris, pH 7.5, 100 mM KCl, 0.25% glycerol, 1 mM TCEP). Peak fractions were quantified using a NanoDrop 8000 Spectrophotometer (Thermo Scientific) and flash frozen and stored at −80°C.

#### Cryo-EM grid preparation and data collection

Wildtype GeoCas9 complexes were frozen on UltrAuFoil R 1.2/1.3 (Electron Microscopy Sciences) grids using FEI Vitrobot Mark IV set to 8°C with 100% humidity. Grids were glow discharged at 15 mA for 25 s using Pelco easiGLOW. 4 μl of sample was applied to the grids and blotted for 4 s with blot force 8. Micrographs for the wildtype complex were collected on Talos Arctica operated at 200 kV and 36,000x nominal magnification (1.14Å pixel size) in super resolution mode (0.57Å pixel size) on K3 Direct Electron Detector in CDS mode. Movies were collected using SerialEM version 4.0.10^56^ with 50 e/Å^2^ final dose. Micrographs for the mutant complex were collected on Titan Krios G2 operated at 300 kV and 81,000x nominal magnification (0.93Å pixel size) in super resolution mode (0.465Å pixel size) on K3 Direct Electron Detector in CDS mode. Movies were collected using SerialEM version 4.0.19^56^ with 50 e/Å^2^ final dose.

#### Cryo-EM data processing

2,767 movies with the wildtype GeoCas9 complex were collected with the defocus range −0.8 to −2. Movies were processed in CryoSPARC software (Structura Biotechnology) version 4.2.1.^51^ Movies were corrected for beam-induced motion with patch motion and CTF parameters were calculated with patch CTF. After manual curation 2,353 micrographs remained for further analysis. Particle picking was optimized using blob, template, and Topaz picking resulting in the extraction of 1,026,723 particles.^57^ They were subjected to 2D classification, and the best classes were selected leaving 949,346 particles. Particles were re-extracted with recentering and subjected to *ab initio* reconstruction into two classes. Only one class resembled the Cas9 complex and contained 645,337 particles. Particles from this class were re-extracted with recentering again. This *ab initio* class was then refined with re-extracted particles using non-uniform refinement resulting in an anisotropic map. Particles were again re-extracted with recentering and classified using 3D classification into 6 classes in the simple mode. Each class was refined using non-uniform refinement.^58^ After refinement only one class was isotropic, had the best completeness and reached the highest resolution of 3.17Å. The best class contained 117,726 particles and served as a basis for model building.

For the iGeoCas9 complex 7,849 movies were collected with defocus range −0.8 to −2. Data was processed in an analogical way as the wildtype complex. After beam-induced motion correction, CTF estimation and manual curation 5,731 micrographs remained. Particles were picked with the blob picker. 6,498,580 extracted particles were subjected to two rounds of 2D classification and class selection. 1,840,865 selected particles were re-extracted with recentering and used for *ab initio* map reconstruction into two classes. 1,368,998 particles from the good class were re-extracted with recentering again and used for refinement of the good class with non-uniform refinement, which resulted in an anisotropic map. Particles from the refinement job were used for the further classification using 3D classification into 6 classes in the simple mode. Each class was refined with non-uniform refinement and the class which reached the highest resolution of 2.63Å was also isotropic and served as a final map for model building.^58^ The final map was obtained from 228,251 particles.

#### Model building

The initial model of wildtype GeoCas9 was generated by ModelAngelo v1.0.1^54^ and was built to wildtype GeoCas9 map that was resampled to pixel size 0.93, and box size 256. Missing residues in the areas of reduced quality density were filled manually with corresponding regions from the Colabfold v1.4.0^48^ model. The density for the following domains was missing in the sharp map and was omitted in the final wildtype GeoCas9 model: Rec-I, residues 134 to 140; HNH, residues 524–665; RuvC-III, residues 749–755; PI residues, 1067–1087. Nucleic acids were built based on Nme2Cas9-sgRNA-dsDNA (PDB: 6JE3)^26^ as an initial model. The density for the following nucleic acids was missing in the sharp map and was omitted in the final wildtype model: sgRNA (5’−3’) nucleotides 71–75, 106–128, 136–139; TS (5’−3’) 1–4, 49–51; and NTS (5’−3’) nucleotides 1–30, and 51. Wildtype GeoCas9 model went through several rounds of real space refinement in Phenix version 1.19.2–4158 and manual geometry improvement in Coot version 0.9.8.7 resulting in a final model.^49,55,59^

The final wildtype model served as the initial model for iGeoCas9, with the introduction of the iGeoCas9 mutations. The iGeoCas9 map was resampled to pixel size 0.93 A, and box size 256. The density for the additional nucleic acids was missing in the sharp map and was omitted in the final iGeoCas9 model:TS (5’−3’) 5 and 45–48; and NTS (5’−3’) 47–50. Model was refined in Phenix with real space refinement.^59^ Geometry was improved manually in Coot and Phenix refinement was repeated to obtain the final iGeoCas9 model.^49,55,59^

#### Electrostatic map generation

Electrostatic maps were generated using the default setting for the Coulombic electrostatic coloring function in ChimeraX v1.6.1.^11^ Map default palette options are assigned as follows: red (−10), white (0), and blue (10).

#### In vitro cleavage assays

For dsDNA cleavage assays comparing activity of non-cognate PAMs, target strands were labeled on the 5’ end with 6-FAM for visualization. DNA substrates were annealed in molecular grade water by heating to 94°C for 2 minutes and slowly cooling to room temperature on a ProFlex PCR system. Substrates included PAM 5’-N_4_CAAA target (doARE784 and doARE785) and non-target (doARE787 and doARE811) DNA, PAM 5’-N_4_CAGA-3’ target (doARE801 and doARE802) and non-target (doARE809 and doARE810) DNA, PAM 5’- N_4_GCAA-3’ target (doARE797 and doARE798) and non-target (doARE805 and doARE806) DNA, and PAM 5’- N_4_TAAA-3’ target (doARE799 and doARE800) and non-target (doARE807 and doARE808) DNA. sgRNA (roARE062) was annealed in 1 × annealing buffer (10 mM Tris, pH 7.5, 20 mM KCl, 1.5 mM MgCl_2_) by heating at 80°C for 2 minutes then placing it on ice. GeoCas9 was incubated with sgRNA (1:1.3) in 1 × cleavage buffer (20 mM Tris-HCl, pH7.5, 100 mM KCl, 5 mM MgCl_2,_ 1 mM TCEP, 5% (w/v) glycerol) at room temperature for 30 min followed by 5 min at 37°C for RNP formation. The final concentration of GeoCas9 RNP was 100 nM and FAM-labeled substrates was 20 nM. Cleavage reactions were initiated by mixing GeoCas9 RNP and FAM-labeled substrate on a 37°C thermoblock. Sample fractions were collected at 0 sec, 30 secs, 1 min, 2.5 min, 5 min, 10 min, 30 min, 60 min, 120 min and mixed with 2 × quench buffer (94% (v/v) formamide, 30 mM EDTA, 400 mg/mL heparin, 0.2% SDS, and 0.025% (w/v) bromophenol blue) to stop the reaction. A substrate only sample represents the “0” fraction. Quenched samples were heated at 95°C for 2 minutes and resolved on a denaturing PAGE gel (12% acrylamide:bis-acrylamide 19:1, 7 M urea, 1X TBE). Samples were visualized using an Amersham Typhoon phosphorimager (GE Healthcare) at 550 V and the.gel image files were quantified using Cytiva ImageQuantTL 10.1.

DNA melting and reduced magnesium *in vitro* cleavage assays were performed with a few differences. In addition to substrates with PAM 5’-N_4_CAAA-3’, the DNA melting assay also included PAM 5’-N_4_GAAA-3’ target (doARE791 and doARE792) and non-target (doARE793 and doARE794) linear substrates, or 2bp mismatch target (doARE791 and doARE803) and non-target (doARE793 and doARE804) DNA. All proteins (wildtype GeoCas9, iGeoCas9, GeoCas9(R1) and GeoCas9(KGR)) contained NLS tags. For reduced magnesium cleavage assays, final cleavage buffer MgCl_2_ concentration is 5 mM, 1mM, 0.1 mM or 0.01 mM, as indicated in the results. Only substrates with PAM 5’-N_4_CAAA-3’ were tested.

#### PAM depletion assays

PAM depletion assays were performed with purified iGeoCas9 and wildtype GeoCas9. RNPs were formed as instructed above (see methods *in vitro* cleavage assays) using 1.25 × the amount of sgRNA to Cas9. Samples included both targeting and non-targeting guides for iGeoCas9 and wildtype Cas9. An “untreated” control was processed alongside in which no RNP was added to the reaction. Libraries were constructed with PAM 5’-TTTTNNNN-3’ downstream of the spacer on the non-target strand, where (N) represents randomized nucleotides. A 206 bp library fragment was generated using overlap PCR with 4 primers (doARE781, doARE782, doARE783, doARE793), and included NexteraXT adapter overhang sequences (*Illumina 16S Metagenomic Sequencing Library Preparation* protocol, Part # 15044223 Rev. B) flanking the target region 20 bp (3’) downstream and 98 bp (5’) upstream of the target sequence. 500 nM of Cas9 RNP was incubated with a 100 nM library for 2 hours at 37°C. Library DNA was purified using 0.8 × AMPure XP beads (Beckman Coulter) and washed three times with 80% ethanol. DNA bound beads were air dried and resuspended in 10 mM Tris-HCl, pH 8.5. Non cleaved library fragments containing both adapter sites were preferentially amplified using Q5 High-Fidelity DNA Polymerase and indexed with NextraXT adapters using 12 cycles of PCR, producing a final library size of 279 bp. PCR products were cleaned with AMPure XP beads as instructed previously. Libraries were quantified using KAPA Library qPCR Quantification Kit (Kapa Biosystems) on a BIO-RAD CFX96 Real-Time System. Libraries were then normalized to 10 nM, pooled, and submitted to the Innovative Genomics Institute (IGI) Next Generation Sequencing (NGS) Core for 100 × sequencing coverage on the NextSeq 2000 (Illumina).

#### NGS analysis for PAM depletion assays

PAM specificity was characterized from FASTQs using a custom Python script. Briefly, regular expressions were used to extract four-nucleotide PAMs. PAM frequencies were calculated and normalized to the sequencing depth of each sample, then the log_2_-fold-change relative to frequencies in the untreated library was determined. Significantly depleted PAMs were defined as those exceeding the 99.9999% confidence interval for maximum log_2_-fold-change depletion in the non-targeting samples. Sequence logos were generated from significant PAMs with Logomaker version 0.8.^53^

#### Monitoring of R-loop formation via 2-aminopurine

DNA duplexes were designed so that 2-aminopurine (2AP) is on the NTS.^24^ There were 3 substrates total with 2 sequential 2AP molecules in positions 1&2, 7&8, 19&20 from the PAM sequence. DNA (doARE825 with doARE824 or doARE826 or doARE828) was annealed at a 1:1.2 molar ratio in (50 mM Tris, pH 7.5, 100 mM NaCl) by heating to 95°C for 3 minutes and then cooling to room temperature over 45 minutes. To prepare the ternary complex, sgRNAs (roARE063 and roARE064) were heated to 95°C for 1 min in ME buffer (10 mM MOPs, pH 6.5 and 1 mM EDTA). To the sgRNA was added 1X reaction buffer (20 mM Tris, pH 7.5; 100 mM KCl, 5% glycerol, with 0.1 mM MgCl_2_ or 5 mM MgCl_2_) before adding Cas9 to assemble sgRNA/GeoCas9 at a 1:1.25 molar ratio in 1X reaction buffer. The GeoCas9/sgRNA mixture was incubated at room temperature for 30 minutes. The reaction was initiated by the addition of the GeoCas9/sgRNA mixture to duplex DNA containing 2-aminopurine at a final molar ratio of 5:1 in 1X reaction buffer at room temperature in a black 384 well plate. The final assembled reaction contained 1 μM 2AP-containing DNA and 5 μM binary complex. Fluorescence emission (λ_em_ 370 nm, λ_ex_ 320 nm) for each reaction was recorded every 20 seconds on a Cytation 5 plate reader (Biotek, software Gen v3.04).

#### HEK293T editing assays

HEK293T EGFP reporter cells were seeded in 96-well plates (20k cell/well) and transfected 24 h later at ~70% confluency according to the manufacturer’s protocol with lipofectamine 3000 (Thermo Fisher Scientific) and 100 ng (50 ng used for dose-limiting conditions) of plasmid DNA encoding the wildtype or engineered Nme2Cas9 and sgRNA. DNA target sequences include guide 1, ggtggtcacgagggtgggccagg; guide 2, agcactgcacgccataggtcagg; guide 3, gggtggtcacgagggtgggccag; guide 4, cctgacctatggcgtgcagtgct; guide 5, ccataggtcagggtggtcacgag; guide 6, tgcacgccataggtcagggtggt; and guide 7, acgccataggtcagggtggtcac. 48 h post-transfection, HEK293T EGFP reporter cells were subjected to selection with 2 μg/mL puromycin in cell culture media for 48 h. Cell culture media was refreshed to exclude puromycin. Cells were collected in 96-well round bottom plates after trypsinization for flow analysis on an Attune NxT Flow Cytometer with an autosampler. Samples were run in biological quadruplicates.

Gene-editing experiments targeting endogenous sites by different Cas9 editors (WT-Nme2Cas9, WT-GeoCas9, Nme2Cas9(C-NR), iNme2Cas9, iGeoCas9, WT-SpyCas9) with HEK293T cells were performed following the same transfection protocol. DNA target sequences included EMX1 guide 1, tccctcattccatgtatcatgct; EMX1 guide 2, gcgttcttcctcccttatgcagt; AAVS1 guide 1, aatatcaggagac taggaaggag; and AAVS1 guide 2, cctccttcctagtctcctgatat. Edited cells were collected, lysed, and subjected to amplicon preparation for next-generation sequencing.

Potential off-targets were identified for two target sites (*EMX1* guide 1 and *AAVS1* guide 2) using Cas-OFFinder (v3.0.0b3)^28^ and the human reference genome GRCh38.p14. Parameters included ≤ 1 bulge, ≤5 mismatches and specific PAMs for each Cas nuclease: WT-GeoCas9 (5’-N_4_CWAA-3’), iGeoCas9 (5’-N_4_CNNN-3’), WT-Nme2Cas9 (5’-N_4_CC-3’), and iNme2Cas9 (5’-N_4_CH-3’). A minimum of 7 off-target sites were PCR-amplified and purified with SPRIselect beads (Beckman Coulter) before NGS library preparation (Berkeley’s IGI NGS Core Facility). Sequencing was performed on an Illumina MiSeq with 23300 bp paired-end reads, to an average depth of ~400,000 reads. Data were processed using Fastp (v0.23.4)^52^ and CRISPResso2 (v2.2.6)^50^ for indel quantification, with a 50% minimum alignment score and two biological replicates when available.

For engineered SpyCas9 EGFP editing assay, 200k HEK293T EGFP cells were nucleofected with 10, 1, or 0.25 pmol pre-assembled RNP (with equal pmol ssDNA enhancer) with program codes of CM-130, according to the manufacturer’s instructions. Lonza SF buffer was used for the preparation of nucleofection mixtures (with a total volume of 20 μl). 10% of the nucleofected cells were transferred to 96-well plates, and the cells were split with a ratio of 5:1 after 3 days. Cells were harvested for analysis after further incubation at 37°C for 3 days. Cells were analyzed on the Attune NxT Flow Cytometer with an autosampler as described above. Samples were run in biological quadruplicates.

### QUANTIFICATION AND STATISTICAL ANALYSIS

For *in vitro* cleavage time course experiments (Figures 3B, C, and 5A) kinetic analyses of substrates were performed in biological replicates (n=3). Using denaturing PAGE, cleaved fractions were determined by dividing the cleaved target strand density by the total density (cleaved + uncleaved) for each sample. The mean was calculated as the sum of the biological replicates divided by the number of the biological replicates. Dispersion among biological replicates for each timepoint is represented as a standard deviation (SD) error bar. Data was quantified in Prism version 9.5.1 and was fit to a curve generated by the formula Y = Ymax *(1 – exp(−k * t)) where Y_max_ is the pre-exponential factor, k is the rate constant k_obs_, and t is the reaction time in minutes. If Ymax ≤ 0.05, k_obs_ is not determined “nd”.

For 2AP experiments (Figure 5D), using Prism version 9.5.1 data was fit to the equation $\text{Y=Y0+Ymax*}\left(1-\hat{e}\left(-\text{k}*\text{t}\right)\right)$ where Y is fluorescence at time (t), Ymax is the fitted reaction endpoint, Y_0_ is the average value of a corresponding blank (binary complex with non-targeting gRNA added to 2AP containing dsDNA), and k is the observed rate constant k_obs_. Each reaction was carried out in triplicate where the k_obs_ reported is the average of each replicate ± standard deviation. If Y_max_ ≤ 100, k_obs_ is not determined “nd”.

Comparison of on-target editing (Figure S6A) was performed in quadruplicate and off-target editing comparisons (Figure S6B) were performed in duplicate, where possible. For on-target editing, the average editing efficiency for each site was plotted with standard-deviation error bars using Prism version 9.5.1. For off-target editing, the average editing efficiency for each site was plotted with individual data points represented as dots using Prism version 9.5.1.

## Supplementary Material

Table S3. Recombinant DNA, related to STAR Methods.

Table S2. Oligonucleotides, related to STAR Methods.

Table S1. Cryo-EM data collection, 3D reconstruction, model refinement, and validation, related to Figure 1.

1

## Figures and Tables

**Table:** KEY RESOURCES TABLE

REAGENT or RESOURCE	SOURCE	IDENTIFIER
Bacterial and virus strains		
*E.coli* BL21(DE3) Competent	New England Biolabs	Cat#C2527H
*E.coli* 5-alpha Competent cells	New England Biolabs	Cat#C2987H
Chemicals, peptides, and recombinant proteins		
2xYT media	Sigma-Aldrich	Cat#Y2377
Ampicillin	Research Products International	Cat#50-550-391
isopropyl b-D-thiogalactoside (IPTG)	Goldbio	Cat#I-902
Tris Hydrochloride (Tris-HCl)	Fisher BioReagents	Cat#BP153-500
Imidazole	Sigma-Aldrich	Cat#IX0005
Tris (2-carboxyethyl) phosphine hydrochloride (TCEP)	Sigma-Aldrich	Cat#C4706
Sodium chloride	JT Baker	Cat#4058
phenylmethylsulfonyl fluoride (PMSF)	Thermo Scientific	Cat#36978
Pierce Human Rhinovirus 3C Protease	Thermo Fisher Scientific	Cat#88946
HEPES	Sigma	Cat#PHG0001
Potassium chloride	Sigma-Aldrich	Cat#P9541
Sodium Acetate	Invitrogen	Cat#AM9740
Molecular grade water	Invitrogen	Cat#AM9939
Acrylamide/Bis 19:1, 40%	Fisher	Cat#10695785
UREA	Sigma	Cat#u5378
Formamide	Fisher Scientific	Cat#BP227-500
Heparin	Fisher Scientific	Cat#BP2425
Bromophenol blue	Fisher Scientific	Cat#AC403140050
Q5 High-Fidelity DNA Polymerase	NEB	M0491
MOPS	Millipore Sigma	Cat#69947
EDTA	Fisher chemical	Cat#S311500
Tris	Invitrogen	Cat#AM9855G
Potassium chloride	Sigma-Aldrich	Cat#P9541
glycerol	Fisher BioReagents	Cat#BP229
Magnesium chloride	Sigma-Aldrich	Cat#M2670
Lipofectamine 3000	Thermo Fisher Scientific	Cat#L3000001
Critical commercial assays		
Nextera XT Index Kit v2 set A	Illumina	Cat#FC-131-2001
KAPA Library Quantification Kit (Illumina) ROX Low	Roche	Cat#KK4873
Deposited data		
Coordinates of iGeoCas9-sgRNA-dsDNA ternary complex	This paper	PDB: 8UZB
Cryo-EM density map of iGeoCas9-sgRNA-dsDNA ternary complex	This paper	EMDB: EMD-42838
Coordinates of GeoCas9-sgRNA-dsDNA ternary complex	This paper	PDB: 8UZA
Cryo-EM density map of GeoCas9-sgRNA-dsDNA ternary complex	This paper	EMDB: EMD-42837
Original code for NGS analysis	This paper	Zenodo: https://zenodo.org/doi/10.5281/zenodo.10774449
Sequencing data	This paper	NCBI BioProject: PRJNA1077744
Experimental models: Cell lines		
HEK293T EGFP reporter cells	University of California Berkeley Cell Culture Facility	N/A
Oligonucleotides		
Oligonucleotides for *in vitro* cleavage assays, 2AP fluorescent assays, and NGS library preparation, and SpyCas9 editing sgRNA, see Table S2.	N/A	N/A
Recombinant DNA		
Plasmids for protein purification and gene editing studies, see Table S3.	N/A	N/A
Software and algorithms		
Cas-OFFinder version 3.0.0b3	Bae et al.^28^	https://github.com/snugel/cas-offinder
ChimeraX version 1.6.1	Pettersen et al.^11^	https://www.cgl.ucsf.edu/chimerax/download.html
Colabfold v1.4.0	Mirdita et al.^48^	https://github.com/sokrypton/ColabFold
Coot Version. 0.9.8.7	Emsley and Cowtan^49^	https://www2.mrc-lmb.cam.ac.uk/personal/pemsley/coot/
CRISPResso2 version 2.2.6	Clement et al.^50^	https://github.com/pinellolab/CRISPResso2
CryoSPARC version 4.4	Punjani et al.^51^	https://cryosparc.com/download
Cytiva ImageQuantTL 10.1	Cytiva Life Sciences	https://www.cytivalifesciences.com/en/us/shop/protein-analysis/molecular-imaging-for-proteins/imaging-software/imagequant-tl-10-2-analysis-software-p-28619
Fastp version 0.23.4	Chen et al.^52^	https://github.com/OpenGene/fastp
Logomaker version 0.8	Tareen and Kinney^53^	https://logomaker.readthedocs.io/en/latest/
ModelAngelo version 1.0.1	Jamali et al.^54^	https://github.com/3dem/model-angelo
Phenix version 1.19.2–4158	Liebschner et al.^55^	https://phenix-online.org/download/
SerialEM version 4.0.10	Mastronarde^56^	https://bio3d.colorado.edu/SerialEM/download.html
Other		
Krios G2 300kV CryoTEM	Thermo Fisher Scientific	N/A
Talos Arctica 200kV CryoTEM	Thermo Fisher Scientific	N/A
NextSeq 2000	Illumina	N/A
MiSeq	Illumina	N/A
Biotek Cytation 5 cell imaging multi-mode reader	Agilent	N/A
Attune NxT Flow Cytometer with an autosampler	Thermo Fisher Scientific	N/A
Ni-NTA Agarose	Qiagen	Cat#30210
Dialysis Cassette 10K MWCO	Thermo Fisher Scientific	Cat#66380
Millex-GP 0.22 uM filter unit	MilliporeSigma	Cat#SLGP033RS
Q500 Sonicator	Sonics & Materials, Inc.	N/A
MBPTrap HP column	Cytiva	Cat#28918779
NanoDrop 8000 Spectrophotometer	Thermo Fisher Scientific	N/A
Amicon Ultra Centrifugal Filter, 3kDa MWCO	MilliporeSigma	Cat#UFC900308
UltrAuFoil R 1.2/1.3	Electron Microscopy Sciences	Cat# Q350AR13A
FEI Vitrobot Mark IV	Thermo Fisher Scientific	N/A
Pelco easiGLOW	Ted Pella	N/A
Amersham Typhoon phosphorimager	GE Healthcare	N/A
AMPure XP Bead-Based reagent	Beckman Coulter	Cat#A63881
BIO-RAD CFX96 Real Time System	BIO-RAD	N/A
ProFlex PCR system	Thermo Fisher Scientific	N/A
Superdex 200 Increase 10/300 GL	Cytiva	Cat#28990944

## Highlights

- iGeoCas9’s enhanced genome-editing activity is driven by three WED-domain mutations
- The WED-domain mutations expand enzyme PAM preference through increased DNA unwinding
- iGeoCas9 has expedited R-loop formation in magnesium-restricted environments
- Rationally engineering the WED domain of CRISPR-Cas genome editors can boost their activity
