# Supplementary material for: Rapid DNA unwinding accelerates genome editing by engineered CRISPR-Cas9
Source: Cell. Author manuscript; Available in PMC 2024 Dec 19. (PMC11658890; doi:10.1016/j.cell.2024.04.031)
Supplement: Table S1. Cryo-EM data collection, 3D reconstruction, model refinement, and validation, related to Figure 1. [file NIHMS2038436-supplement-Table_S1__Cryo-EM_data_collection__3D_reconstruction__model_refinement__and_validation__related_to_Figure_1_.pdf]

Cell, Volume *187*

Supplemental information

Rapid DNA unwinding accelerates genome editing by engineered CRISPR-Cas9

Amy R. Eggers, Kai Chen, Katarzyna M. Soczek, Owen T. Tuck, Erin E. Doherty, Bryant Xu, Marena I. Trinidad, Brittney W. Thornton, Peter H. Yoon, and Jennifer A. Doudna

**Data collection**		
sample	Wildtype GeoCas9	iGeoCas9
Electron Microscope	FEI Talos Arctica	Titan Krios G2
Electron Detector	K3 Direct Electron Detector	K3 Direct Electron Detector
Magnification	36,000	81,000
Voltage (kV)	200	300
Electron dose (e-/Å^2)	50	50
Defocus range (μm)	-0.8 to 2	-0.8 to 2
Pixel size (Å)	0.57	0.465
**3D reconstruction**		
sample	Wildtype GeoCas9	iGeoCas9
Raw images	2,767	7,849
Initial particles	1,026,723	6,498,580
Final particles	117,726	228,251
Map resolution (Å)	3.17	2.63
FSC threshold	0.143	0.143
**Model refinement**		
sample	Wildtype GeoCas9	iGeoCas9
Initial model used	*Ab initio* ModelAngelo model<br>*Ab initio* Colabfold model	*Ab initio* ModelAngelo model<br>*Ab initio* Colabfold model
Model resolution	3.1	2.6
FSC threshold	0.143	0.143
Model composition<br>Non-hydrogen atoms<br>Protein residues<br>Nucleotide<br>Ligands	 11072<br>910<br>171<br>0	 10882<br>910<br>162<br>0
B factors (mean, Å^2)<br>Protein<br>Nucleotide	 65.91<br>92.98	 54.29<br>69.52
R.m.s deviations<br>Bond length (Å)<br>Bond angles (°)	 0.003<br>0.509	 0.003<br>0.528
**Validation**		
sample	Wildtype GeoCas9	iGeoCas9
MolProbity score	1.71	1.55
Clash score	7.12	5.68
Rotamer outlier (%)	0	0
Ramachandran statistics (%)-<br>Favored<br>Allowed<br>Outlier	 95.45<br>4.55<br>0	 96.34<br>3.66<br>0
Rama-Z score, whole<br>(r.m.s Rama-Z)	-0.07<br>0.28	0.50<br>0.28
Map CC (box)	0.76	0.67
Map CC (mask)	0.82	0.78

Table S1. Cryo-EM data collection, 3D reconstruction, model refinement and validation, related to Figure 1
