# Supplementary material for: Rapid DNA unwinding accelerates genome editing by engineered CRISPR-Cas9
Source: Cell. Author manuscript; Available in PMC 2024 Dec 19. (PMC11658890; doi:10.1016/j.cell.2024.04.031)
Supplement: 1 [file NIHMS2038436-supplement-1.pdf]

Supplemental figures

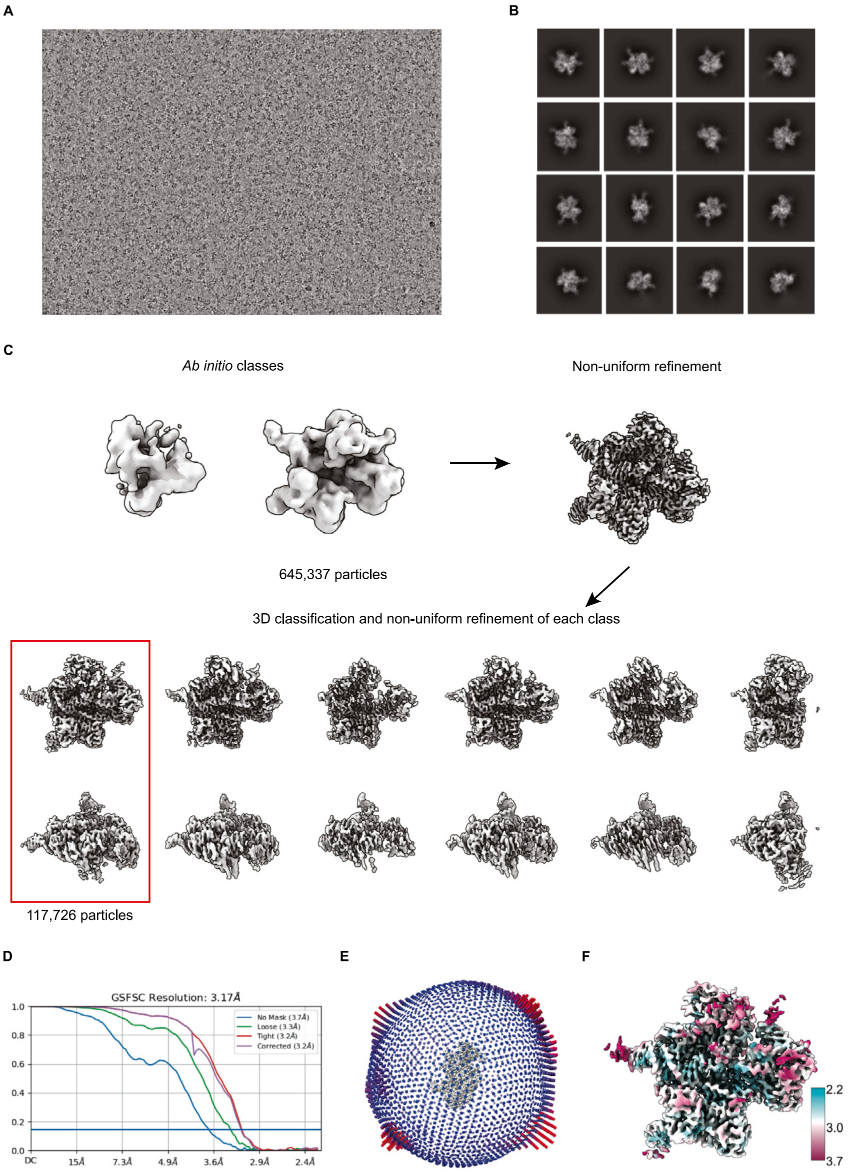

Figure S1. Cryo-EM workflow for wild-type GeoCas9, related to Figures 1 and 2

(A) Example cryo-EM image after beam-induced motion correction.

(B) A subset of selected 2D class averages.

(C) Cryo-EM data processing in cryoSPARC v.4.4.[51] *Ab initio* classes are visualized at 0.2 contour level and refined classes at 0.27 contour level. Red box highlights the final map.

(D) Gold standard Fourier shell correlation (FSC) curves from the final round of non-uniform refinement in cryoSPARC v.4.4.[58]

(E) Particle orientation distribution.

(F) Local resolution map for final map calculated in cryoSPARC v.4.4[51] with threshold 0.143 and displayed in ChimeraX v.1.6.1[11] with dust removal size 5 and contour level 0.27.

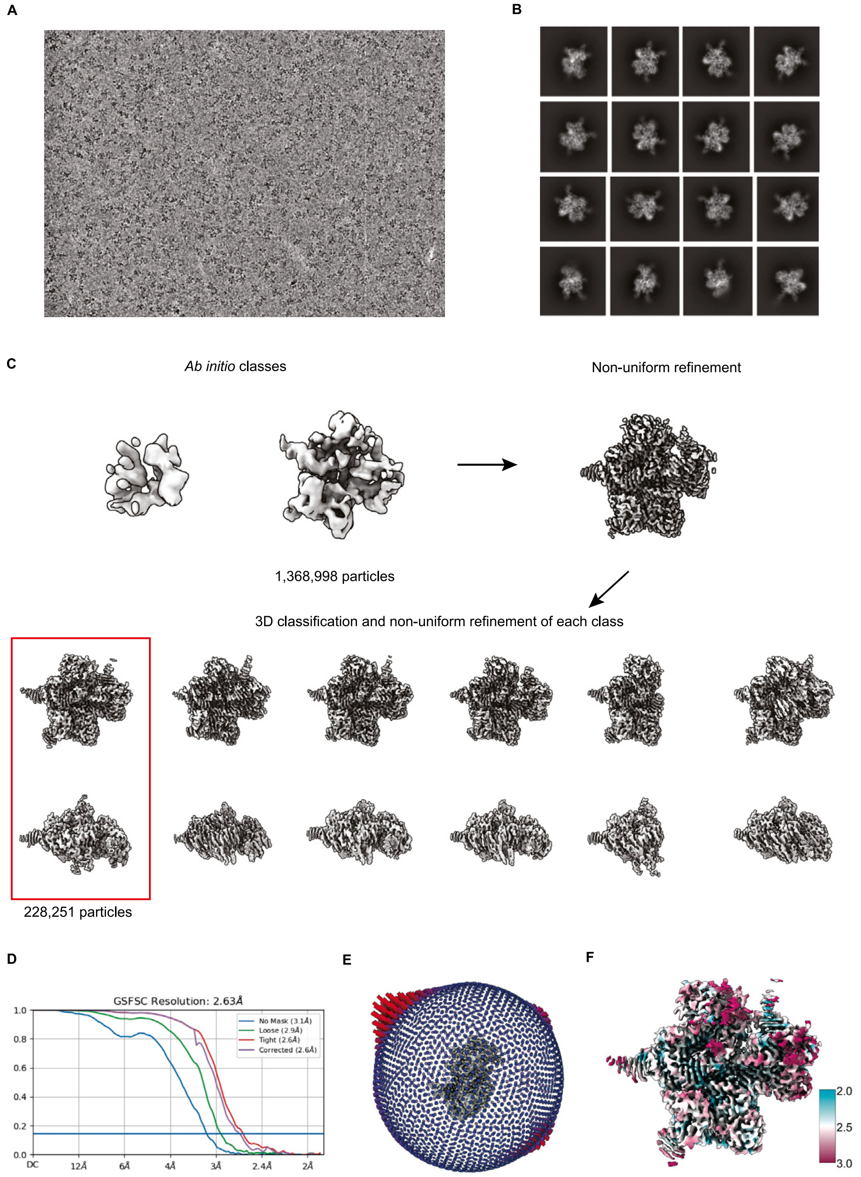

Figure S2. Cryo-EM workflow for iGeoCas9, related to Figures 1 and 2

(A) Example cryo-EM image after beam-induced motion correction.

(B) A subset of selected 2D class averages.

(C) Cryo-EM data processing in cryoSPARC v.4.4.[51] *Ab initio* classes are visualized at 0.2 contour level and refined classes at 0.27 contour level. Red box highlights the final map.

(D) Gold standard FSC curves from the final round of non-uniform refinement in cryoSPARC v.4.4.[58]

(E) Particle orientation distribution.

(F) Local resolution map for final map calculated in cryoSPARC v.4.4[51] with threshold 0.143 and displayed in ChimeraX v.1.6.1[11] with dust removal size 5 and contour level 0.27.

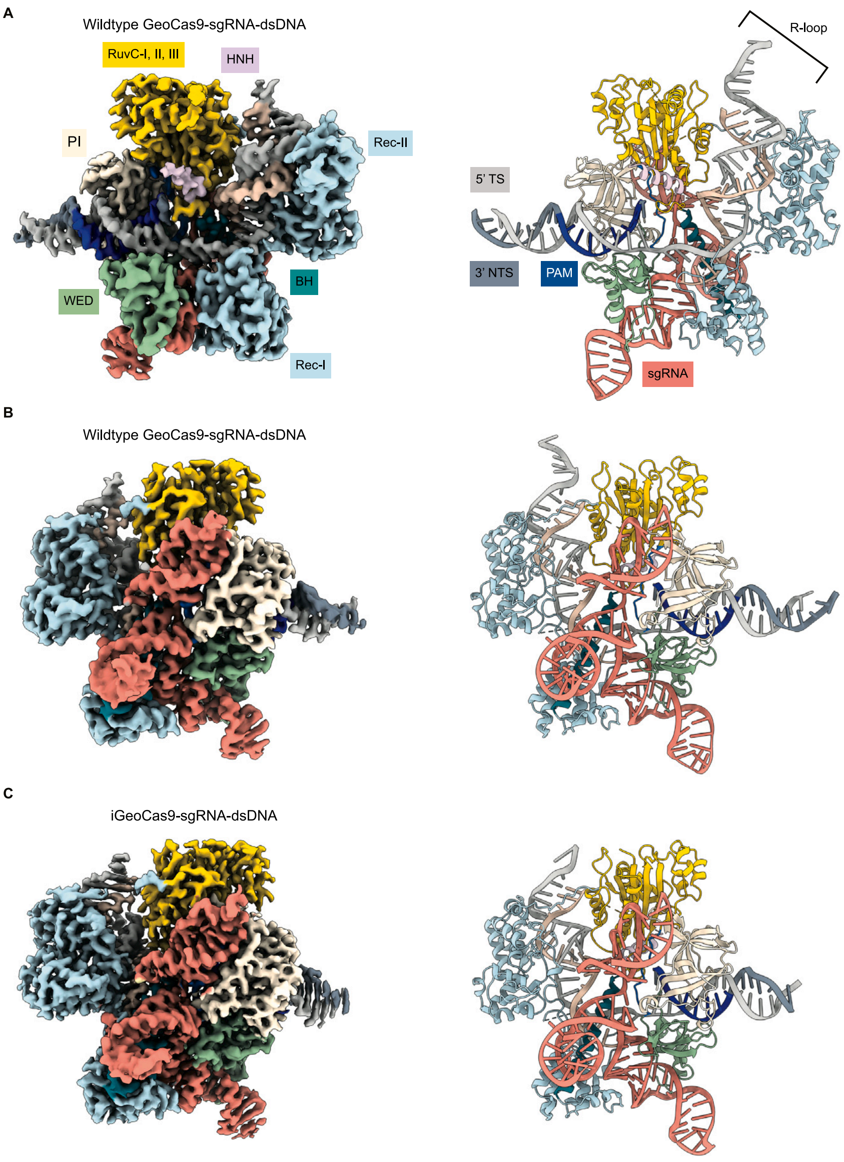

Figure S3. Cryo-EM density maps and models, related to Figures 1 and 2

(A) Cryo-EM density map of wild-type GeoCas9-sgRNA-dsDNA complex (left) at 3.17 Å resolution with domains labeled as in Figure 1A. Wild-type GeoCas9 model complex (right) with nucleic acids labeled as in Figure 1D. BH, bridge helix; WED, wedge; PI, PAM interacting; TS, target strand; NTS, non-target strand; PAM, protospacer adjacent motif; sgRNA, single guide RNA.

(B) Cryo-EM density map (left) and model (right) of wild-type GeoCas9-sgRNA-dsDNA complex rotated 180° from part (A). Domains colored as in Figure 1A. Nucleic acids colored as in Figure 1D.

(C) iGeoCas9sgRNA-dsDNA complex density map (left) at 2.63 Å resolution and corresponding model (right). Both are rotated 180° from Figure 1. Domains colored as in Figure 1A. Nucleic acids colored as in Figure 1D.

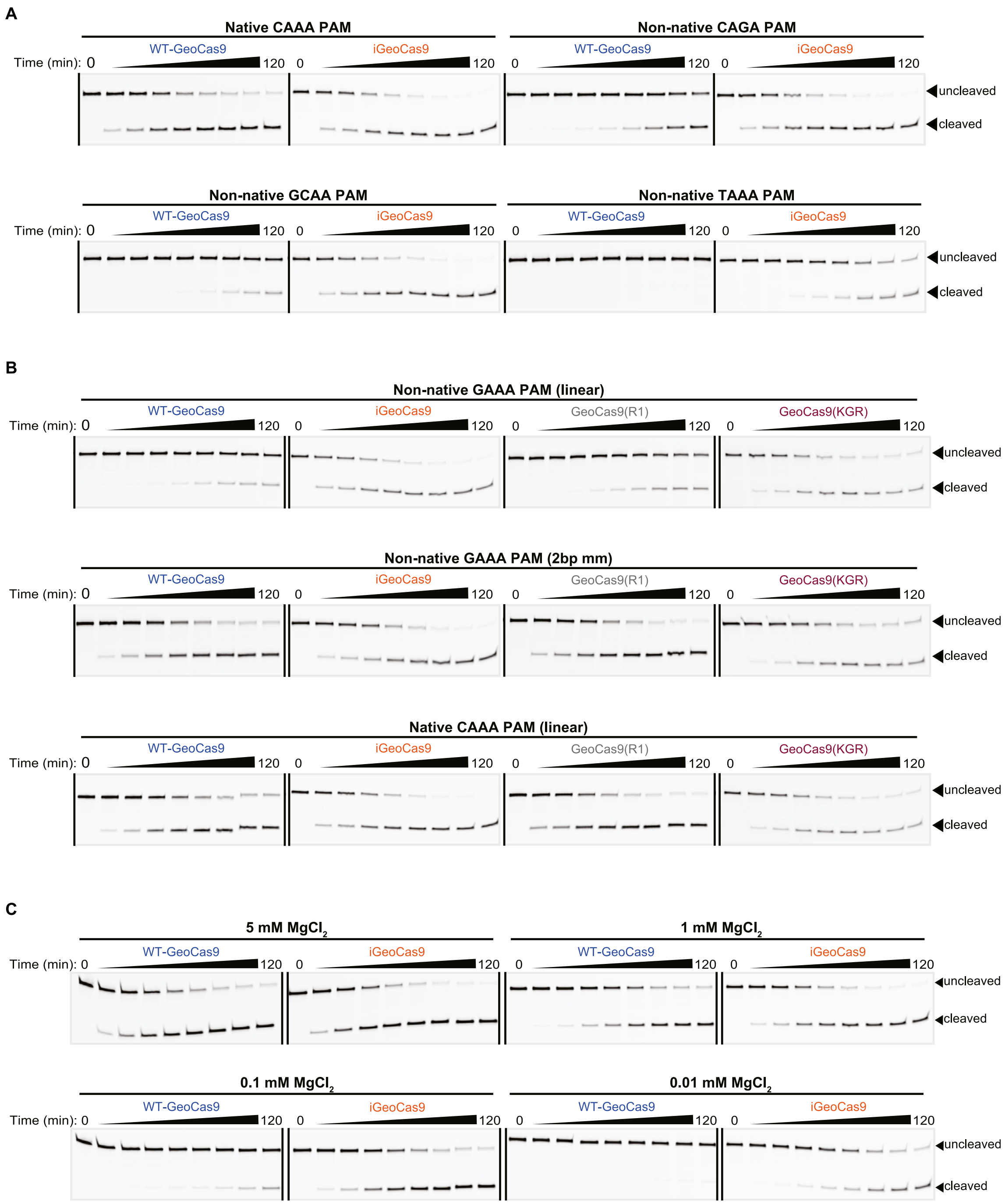

Figure S4. ***In vitro*** **cleavage kinetics of wild-type GeoCas9, iGeoCas9, GeoCas9(R1), and GeoCas9(KGR), related to Figures 3, 4, and 5**

In vitro dsDNA cleavage was determined using denaturing PAGE. 60 nt substrates are 5′ 6-FAM labeled. Fractions were collected at 0 s, 30 s, 1 min, 2.5 min, 5 min, 10 min, 30 min, 1 h, and 2 h. "0" fractions contain substrate only. Images rendered in Image Lab 6.1 (Bio-Rad) and cropped. Black lines indicate neighboring irrelevant lanes were spliced out.

(A) iGeoCas9 exhibits broader PAM preferences *in vitro* than WT-GeoCas9, related to Figure 3. PAM contained in each substrate listed above gel images.

(B) Thermodynamically unstable substrate mimics WED-domain mutation effects on DNA melting, related to Figure 4. PAM and substrate design listed above gel images.

(C) The impact of $MgCl_2$ on WT-GeoCas9 and iGeoCas9 activity, related to Figure 5. $MgCl_2$ concentrations for each experiment indicated above gel images.

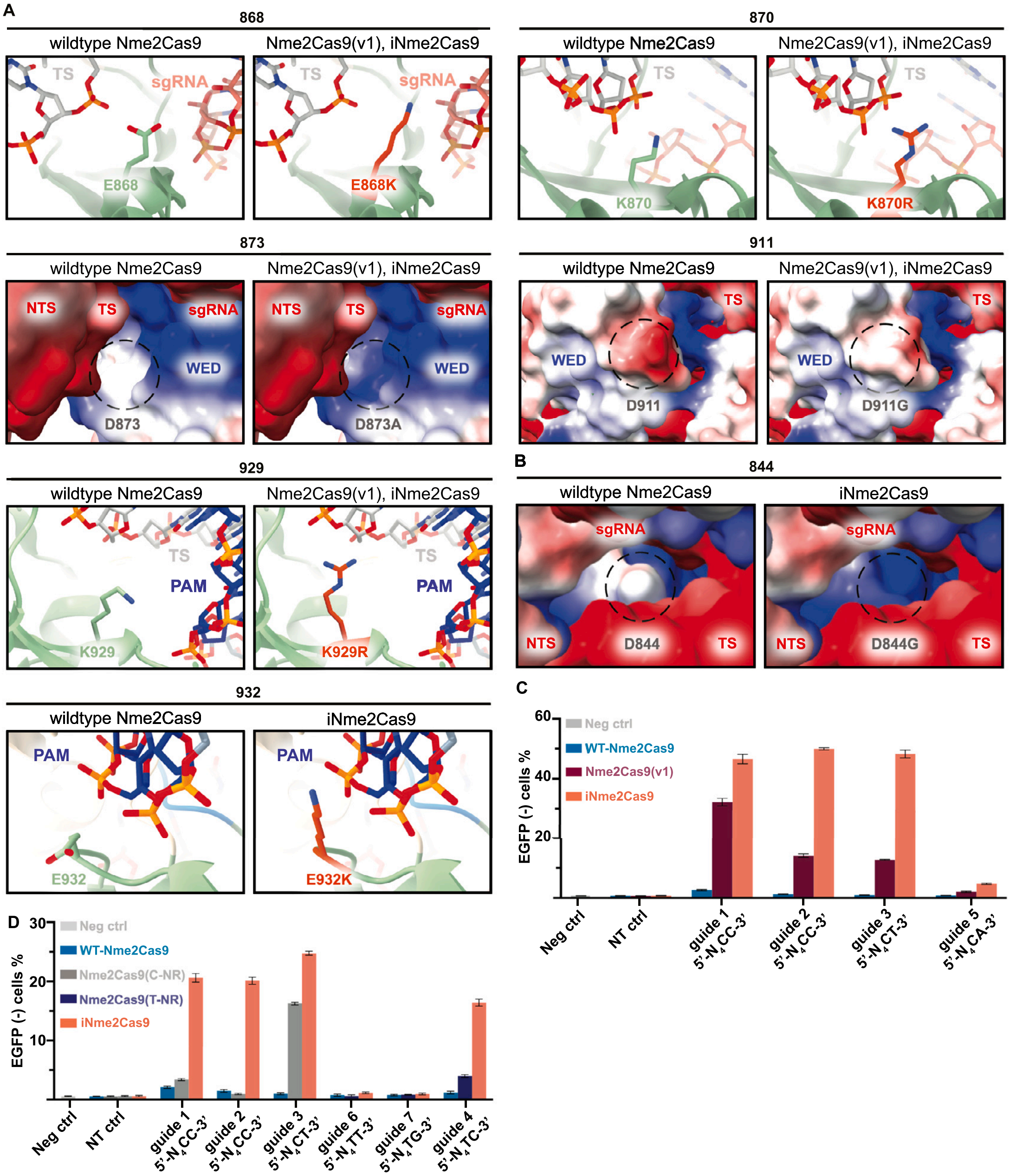

Figure S5. Improved genome editors Nme2Cas9(v1) and iNme2Cas9 compared with wild-type Nme2Cas9, related to Figure 6

(A) Structural comparison of wild-type Nme2Cas9 (PDB: 6jE3)[26] and mutations contained in Nme2Cas9(v1) and iNme2Cas9. Residue number located above images. Nme2Cas9(v1) and iNme2Cas9 mutations were created *in silico* using ChimeraX v1.6.1[11] using the wild-type Nme2Cas9 model. Rotamers were chosen to demonstrate potential DNA interactions. Mutations expected to interact with DNA are represented as sticks and ribbons. Mutations expected to alter protein charge are represented as Coulombic electrostatic potential maps and were calculated using default settings in ChimeraX v1.6.1[11] (red, negative; blue, positive; white, non-polar).

(B) Structural comparison of wild-type Nme2Cas9 (PDB: 6jE3)[26] and mutations contained in iNme2Cas9. Mutations expected to interact with DNA are represented as sticks and ribbons. Mutations expected to alter protein charge are represented as Coulombic electrostatic potential maps and were calculated using default settings in ChimeraX v1.6.1[11] (red, negative; blue, positive; white, non-polar).

(C) HEK293T cell editing by wild-type (WT-)Nme2Cas9, Nme2Cas9(v1), and iNme2Cas9, with 4 different guides ($n = 4$, data are represented as mean ± SD). PAM sequence is below the corresponding samples. Neg ctrl, no treatment control; NT, non-targeting guide control.

(D) HEK293T cell editing by wild-type (WT-)Nme2Cas9, iNme2Cas9, Nme2Cas9(C-NR), and Nme2Cas9(T-NR) with 6 different guides ($n = 4$, data are represented as mean ± SD). The PAM sequence is below the corresponding samples. Neg ctrl, no treatment control; NT, non-targeting guide control.

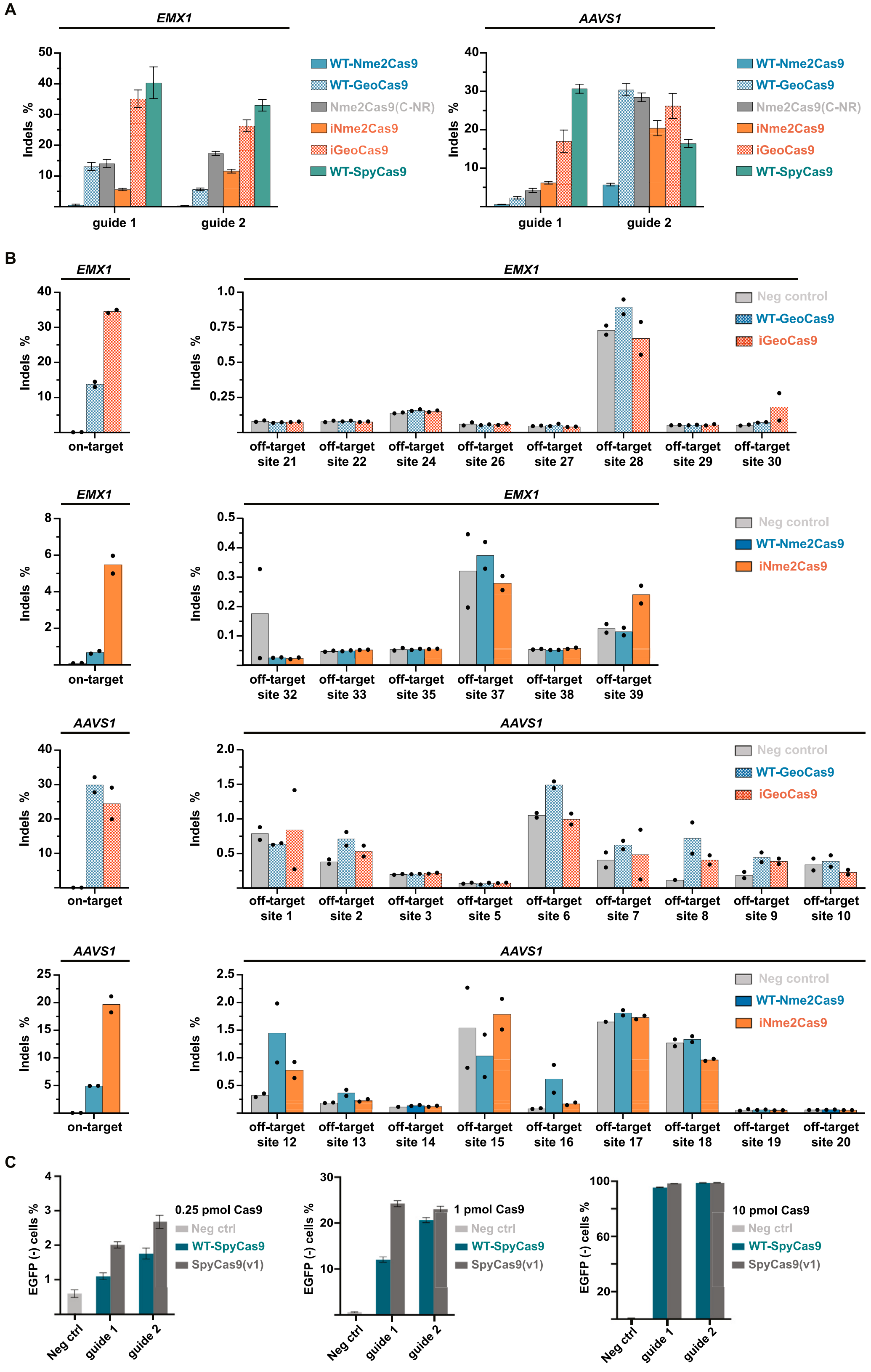

(legend on next page)

CellPress
OPEN ACCESS

Figure S6. A comparison of improved genome editors iNme2Cas9 and iGeoCas9 to WT-Nme2Cas9, Nme2Cas9(C-NR), WT-GeoCas9, and WT-SpyCas9, and preliminary engineering of SpyCas9, related to Figure 6

(A) Editor activity against genes *EMX1* (left) and *AAVS1* (right) in HEK293T cells, each with two different guides ($n = 4$, data are represented as mean ± SD). Editors listed in legend. Indels, insertions, or deletions.

(B) On-target (left) and off-target (right) editor activity against genes *EMX1* (guide 1) and *AAVS1* (guide 2) ($n = 2$, data values represented as individual dots). Gene listed above each graph. % indels on y axis and off-target site on x axis. Editors listed in legend. Indels, insertions, or deletions.

(C) HEK293T cell editing based on nucleofection of SpyCas9 RNPs, including WT-SpyCas9 or SpyCas9(v1) with 2 guides ($n = 4$, data are represented as mean ± SD). Mutations in SpyCas9(v1) relative to WT-SpyCas9 include: G1104R, D1117A, D1125A, and A1285K. Neg ctrl, no treatment control.
